# Ciliary IFT‐B Transportation Plays an Important Role in Human Endometrial Receptivity Establishment and is Disrupted in Recurrent Implantation Failure Patients

**DOI:** 10.1111/cpr.13819

**Published:** 2025-02-06

**Authors:** Haoxuan Yang, Jing Zhang, Fei Yan, Yihong Chen, Yang Wu, Jiaxin Luo, Lian Duan, Juan Zou, Juncen Guo, Jiyun Pang, Andras Dinnyes, Jiuzhi Zeng, Weixin Liu, Chi Chiu Wang, Yi Lin, Xue Xiao, Xiaomiao Zhao, Wenming Xu

**Affiliations:** ^1^ Department of Gynecology and Obstetrics, Joint Laboratory of Reproductive Medicine (SCU‐CUHK) West China Second University Hospital of Sichuan University Chengdu P. R. China; ^2^ Key Laboratory of Birth Defects and Related Diseases of Women and Children Sichuan University, Ministry of Education Chengdu P. R. China; ^3^ Department of Day Surgery West China Second University Hospital of Sichuan University Chengdu P. R. China; ^4^ Department of Reproductive Medicine Sichuan Provincial Maternity and Child Health Care Hospital Chengdu P. R. China; ^5^ Department of Pediatric Pulmonology and Immunology West China Second University Hospital of Sichuan University Chengdu P. R. China; ^6^ Department of Obstetrics Chengdu Jinjiang Hospital for Women and Children Health Chengdu P. R. China; ^7^ Department of Pathology West China Second University Hospital of Sichuan University Chengdu P. R. China; ^8^ West China Hospital of Sichuan University Chengdu P. R. China; ^9^ Department of Physiology and Animal Health, Institute of Physiology and Animal Nutrition Hungarian University of Agriculture and Life Sciences Godollo Hungary; ^10^ BioTalentum Ltd. Godollo Hungary; ^11^ Chinese University of Hong Kong‐Sichuan University Joint Laboratory in Reproductive Medicine The Chinese University of Hong Kong Hong Kong P. R. China; ^12^ Shanghai Jiao Tong University Affiliated Sixth People's Hospital Shanghai China; ^13^ Department of Gynecology and Obstetrics West China Second University Hospital of Sichuan University Chengdu P. R. China; ^14^ Department of Reproductive Medicine, Guangdong Provincial People's Hospital (Guangdong Academy of Medical Sciences) Southern Medical University Guangzhou P. R. China

**Keywords:** cilia sphere, ciliogenesis, endometrial organoid, endometrial receptivity, endometrium, intraflagellar transport

## Abstract

The lack of accurate understanding of cellular physiology and pathophysiology during the WOI constitutes the major obstacle to correct diagnosis and treatment for patients with recurrent implantation failure (RIF). The role of cilia as one of the key organelles in endometrial epithelium has been poorly understood during embryo implantation. In this study, the morphological and molecular changes of endometrial cilia regulated by hormones were demonstrated in endometrial epithelial organoid models. Multi‐omics studies revealed highly relevant cilia‐related activities like cilia movement during endometrial receptivity establishment. Interestingly, both in vitro model and in vivo patient data have shown that the apical part of cilium formed a cilia‐derived spherical structure after hormone stimulation. We also found intraflagellar transport (IFT) train multi‐subunit complex B (IFT‐B) was aggregated in the sphere during the implantation window. Meanwhile mitochondria localization signal increased at the cilia basement. Proteomics and the functional assay showed the deficiency of energy metabolism in RIF patients and cilia formation abnormalities. The abnormal energy supply resulted in the failure of vesicle transport by deficiency of IFT‐B location, ultimately leading to the failure of receptivity establishment. Our study revealed the essential role of endometrial cilia in embryo implantation and indicated that mitochondrial metabolism was crucial for normal ciliogenesis and embryo implantation.

## Graphical abstract

Our results suggested that mitochondria‐accelerated motile cilia intraflagellar transport may be involved in endometrial microenvironment establishment.

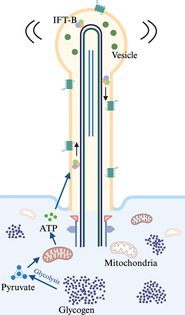

## 1 Introduction

Infertility, based on the clinical definition, refers to failure to achieve a clinical pregnancy after 1 year of routine unprotected sex [1], and to failure of good‐quality embryo transplantation for more than two cycles, patients could be diagnosed with recurrent implantation failure (RIF) [2, 3]. Currently, the human natural pregnancy rate is ~75% at the age of 30 years but it gradually decreases with aging. In addition, for the age group 35–40 years, assisted reproductive technology (ART) can provide resolution in < 30% of infertility cases [4]. For embryo implantation to be successful, an appropriate crosstalk between the endometrium and the embryo is needed. The aetiology of unexplained RIF is often elusive for patients who exclude endometriosis, PCOS and other genital tract inflammation and routine physical examination was normal. There is a lack of personalised methods or accurate biomarkers to evaluate endometrial receptivity and predict the optimal implantation time. Several methods including the Endometrial Receptivity Array (ERA) and Endometrial Receptivity test (ERT) are wildly used in reproductive centres now [5], and they may help subfertility patients to detect optimal WOI for improving IVF‐ET success, though these technologies may not benefit all RIF patients. Furthermore, ERA cannot provide the pathogenesis insight for patients with unexplained implantation failure, and it remains controversial whether ERA can reflect endometrial receptivity by gene detecting [6, 7, 8]. Clearly, a better understanding of the molecular mechanisms is necessary to develop more accurate biomarkers and potential new pharmaceutical targets for RIF patients. Considering that during preimplantation hormone level variations are resulting in dynamic changes in the endometrium, a human tissue‐derived model would be suitable to reproduce these changes and to study them under continuous observation.

Cilia have been shown to play important roles in the female reproductive system [9, 10]. Motile ciliary gene mutations constitute a significant part of female infertility [11]. Cilia can be found in the fallopian tubes where they play an essential role in oocyte pickup [12] and affect laminar flow for transport [13]. However, the role of motile cilia and the underlying regulation in the endometrial lumen has not been described fully. Indirect evidence has shown that over‐activated motile cilia may prevent embryo implantation by liquid flow and cilia disappear at the implantation site [14, 15]. Recent studies, including ours, have shown that mitochondrial metabolic activity plays a critical role in ciliogenesis and ciliary activity. Using the pulmonary epithelial organoid model, we have shown that key metabolic molecules, including NADH, can improve cilia motility through modulating mitochondrial respiratory activity [16]. The research on sperm has found that IFT20 ciliary motor protein participated in mitochondria movement to permit spermatogenesis success [17], and IFT88 is proven to contribute to mitochondrial homeostasis [18]. Recent transcriptome analyses have shown that the motile ciliary gene signature may be a marker of endometrial aging [19]. Several studies have documented that genes related to cilia change significantly during the WOI [20, 21, 22], yet the regulation and functional role of motile cilia during the WOI in the endometrium remains poorly elucidated.

Organoids derived from the endometrium show typical implantation structures after oestrogen combined progesterone stimulation, such as pinopodes and cilia, and similar epithelial protein expression patterns [23, 24, 25, 26]. Metabolomic analyses have shown that intraorganoid fluid is biochemically distinct from extraorganoid fluid [27], indicating that there is active protein/metabolism transport from the basolateral side to the apical side which is consistent with that of the uterus in vivo. Organoids can mimic the human uterine microenvironment and mimic epithelial cell changes during implantation processes. Taking advantage of these new advancements, this study therefore aimed to investigate the changes in the characteristics of ciliated cells of organoids in the secretory stage.

Here, we used endometrial organoids derived from three control women of childbearing age and three RIF patients and simulated the menstrual cycle in vitro by steroid induction to record molecular changes at different phases. RNA sequencing showed distinct ciliary activity increases after hormone stimulation, including the increase in ciliary assembly, ciliary movement and microtubular motor protein activity. Immunofluorescence and electron microscopy results indicated that a novel structure at the tip of the cilia, the cilia‐derived sphere (CDS), may participate in the receptive endometrial microenvironment establishment via IFT‐B transport. Interestingly, our results showed that mitochondria and lysosomes are enriched at the basement membranes of cilia. Furthermore, the ciliary ultramicroproteome results implied that mitochondrial proteins were transported to the ciliary body which then may participate in endometrial receptivity establishment. In RIF organoids, however, defective IFT‐B complex formation and disappearance of the polar distribution of mitochondria were observed. Furthermore, we found that hTSCs spheres that mimic embryos could not be implanted in the endometrial cell layer when treated with a cilia inhibitor. Overall, our result supports the notion that CDS was regulated by hormone stimulation, and IFT‐B complex aggregated in the apex which participated in the construction of the receptive uterine microenvironment promoted by mitochondrial energy supply. Our study may have uncovered a new cilia‐related transport pathway in which the endometrium is involved in the implantation via the CDS structure. The defective CDS formation might be related to RIF in clinical cases.

## 2 Material and Methods

### 2.1 Clinical Sample Collection

All the experimental protocols, including human tissue collection and animal experiments, were approved by the Ethics Committee of West China Second Hospital, Sichuan University (ethical approval number: K2018062), and were based on the World Medical Association's Declaration of Helsinki guidelines. Written informed consent was acquired from each participant before the sample was obtained.

Endometrial biopsies for experiments were collected from West China Second Hospital. A total of three positive samples from RIF patients (aged 22–34 years) who failed to achieve a clinical pregnancy after 1 year of routine unprotected sex and to failure of good‐quality embryo transplantation for more than two cycles seeking surgical treatment for infertility were included in this study, as were three normal control samples from fertile women aged 22–34 years who had natural pregnancy and delivery histories and were seeking a new pregnancy. None of the participants had genital tract inflammation, polycystic ovary syndrome (PCOS), endometriosis or other known factors that could induce failure of implantation by hysteroscopic and laparoscopic surgery.

### 2.2 Primary Cell Isolation and Endometrial Epithelial Organoid Establishment

This protocol was referenced from previously published studies [23]. In brief, endometrial epithelial primary cells were mixed with Matrigel after separation. Thirty‐microliters droplets of Matrigel‐cell suspension were added to a 24‐well plate (1 droplet per well) and incubated at 37°C for 15 min, then the organoid medium was overlaid in each well. The organoids formed after 4–7 days of cultivation and were passaged after they attained a diameter of more than 400 μm.

### 2.3 Hormone Stimulation of Organoids

**FIGURE 1:**
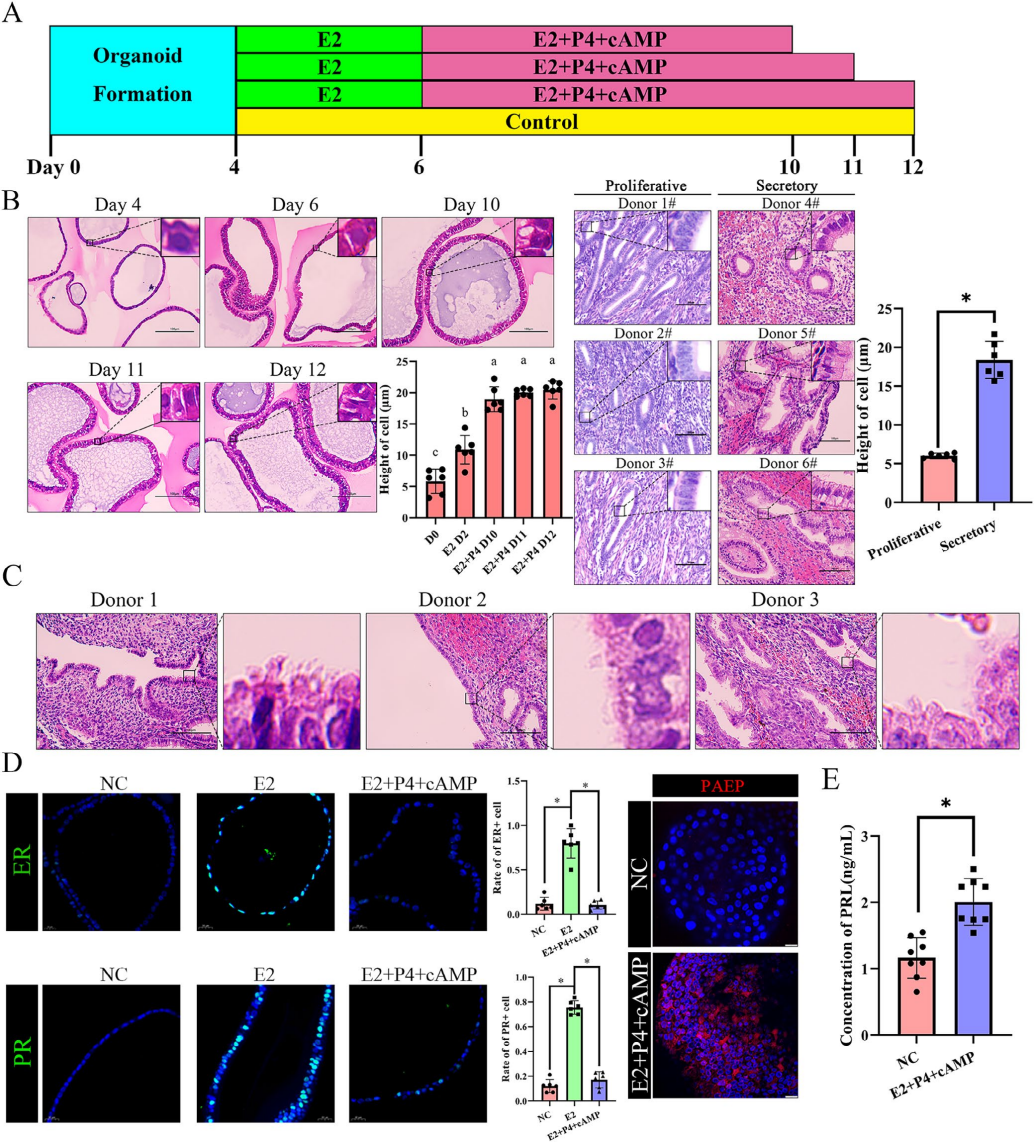
Oestrogen combined with progesterone‐induced organoid transformation to a secretory phase‐like state. (A) Organoids were then treated with either E2 or vehicle as a control for 2 days. Following this, organoids were treated with E2 + P4 + cAMP for a further 4, 5 and 6 days. (B) H&E staining showed cell morphological changes after hormone stimulation at each collection site (D4, D6, D10, D11, D12). The left panel shows the cell morphological changes in endometrial organoids. Scale bars, 100 μm. a, b, c means *p* < 0.05. Small black frame: Partial enlarged detail. The right panel shows the cell morphological changes in endometrial tissue in the proliferation and secretory stage. Scale bars, *upper*: 200 μm; *b*ottom, 100 μm. *means *p* < 0.05. (C) Tissue H&E staining of secretory endometrium checked chamber and cilium appeared in epithelial and glandular cells from different donors. Scale bars, 100 μm. (D) ER and PR nuclear localization was increased in response to E2 and decreased after progesterone addition which in accordance with the in vivo condition. These statistical data of ER+ cell and PR+ cell proportion are shown on the right bar graph. And PAEP expression was significantly increased after progesterone stimulation. Organoids were counterstained with DAPI (blue) to visualise nuclei. Scale bars, D left, 10 μm, D right, 20 μm. The experiment was repeated three times. (E) PRL content increased after progesterone stimulation. *means *p* < 0.05.

As published before [23, 24], the organoids were treated with either 10 nM estradiol (Sigma, E1024, USA) or the vehicle chemical (100% ethanol) as a control for 2 days. Following this, the organoids were treated with either 10 nM E2 or 10 nM E2, 1 μM P4 (Sigma, PHR1589, USA) and 1 μM cAMP (2′‐Odibutyryladenosine 3′,5′‐cyclic monophosphate sodium salt; Sigma, D0627, USA) for a further 4, 5 and 6 days. Samples were collected at each time point (Figure 1A).

### 2.4 HE Staining

Tissue sections of 4 μm were cut from paraformaldehyde‐fixed paraffin‐embedded human endometrial organoids. Before paraffin embedding, the organoids were removed from the Matrigel using Cell Recovery Solution (Corning, 354253, USA), fixed in 4% paraformaldehyde (Biosharp, BL539A, China) and embedded in 1% agarose (Sangon, A620014, China). The sections were dewaxed with xylene and rehydrated with an ethanol gradient (100%, 95%, 85%, 75% and 50%). The slides were set in pure water. Then, haematoxylin staining was performed for 5 min, and after running water washes, the slides were differentiated with 1% hydrochloric acid alcohol. Following 0.5% eosin staining, these slides were sealed with neutral resin after dehydration. The results were captured by optical microscopy (Olympus, BX43, Japan).

### 2.5 RNA Sequencing

Organoids were recovered from Matrigel with cell recovery solution and stored at −80°C. Total RNA was isolated with TRIzol (Ambion, USA) immediately after sample collection from the operating room, and cDNA synthesis was performed using a Takara Bios RR047A complementary DNA (cDNA) synthesis kit. Sequencing libraries were generated and sequenced by Novogene (Beijing, China). GO analysis takes *p*_adj_ < 0.05 as the threshold of significant enrichment.

### 2.6 Proteomics

Proteomics were identified by a tandem mass tag (TMT). Briefly, organoids collection was performed as above for RNA‐Seq and lysed by 4% SDS, after ultrasonic destruction of nucleic acid, the concentration was analysed by bicinchoninic acid (BCA) kit (Thermo, Cat. no. 23225). After enzyme digestion, these peptide fragments were labelled by TMTpro 16plex Isobaric Label Reagent Set (A44520, Thermo). The peptides were re‐dissolved in Solvent A (A: 0.1% formic acid in water) and analysed by Orbitrap Fusion coupled to an EASY‐nanoLC 1200 system (Thermo Fisher Scientific, MA). The raw data of DDA were processed and analysed by SpectroMine3.2 (Biognosys AG, Switzerland) with default settings. The database was UniProt‐ *Homo sapiens* (version 2022, 20,610 entries). The normalisation was calculated from the total intensity of all labels in all quantifiable peptides. Different expressed proteins were selected if their *p* value < 0.05, FC > 1.3, < 1/1.3.

### 2.7 Ultramicroproteome

Ultramicroproteome was identified by 4D Data Independent Acquisition (DLA). Ultrathin sections of organoids (2 mm) were prepared (by HistoCore BIOCUT, Leica) after paraffin embedding, and then these sections were recovered from the water by Frameslide (Leica, 11505151, Germany) followed by drying on a thermal platform at 65°C. After programmed dewaxing and rehydration, samples of the cilia that contain the spheres were collected by Leica microdissection system (Leica, LMD6, Germany). We totally collected 650 cilia from three different organoid origins for subsequent proteomics analysis. Ultramicroproteome was generated and sequenced by Westlake Omics (Hangzhou, China).

### 2.8 RT‐qPCR and Digital PCR

The experiments were performed as previously described [28]. The specific RT‐PCR primers are listed in Table S1. Briefly, total RNA was isolated using TRIzol reagent (Invitrogen) and cDNA was synthesised using the kit (TaKaRa, RR047A, Japan). A real‐time RT‐qPCR assay was performed using a Takara RR820A kit (Takara, Japan) according to the manufacturer's instructions. The relative mRNA expression levels were calculated using the 2^−ΔΔCt^ (Livak) method.

To detect mitochondria DNA copy changes after hormone stimulation in organoids, we used digital PCR. Organoid DNA isolation was performed according to the instruction of the nucleic acid extraction kit (R00301, Biorain, China) and dPCR chip also purchased from Biorain. PCR primers are listed in Table S1.

### 2.9 Immunofluorescence

For paraffin sections, after dewaxing and rehydration, the sections were blocked with 5% BSA (BioFroxx, 4240GR500, China) for 1 h. These slides were incubated in a 4°C refrigerator overnight after being covered with 1% BSA‐diluted primary antibody. Then, the slides were rinsed with sterile PBS three times for 5 min each and incubated with a secondary antibody conjugated with a fluorophore for 1 h at room temperature. After being washed three times, these slides were stained with DAPI combined with an anti‐fluorescence quencher. After these slides were sealed, these results were captured by confocal microscopy (Olympus, FV3000, Japan).

For directed staining, these fixed organoids were washed with ice‐cold PBS three times in 1.5 mL EP tubes for 10 min each time. The tubes were then centrifuged at 800 rpm for 5 min to sediment the organoids and remove the supernatant. These organoids were treated with 1% Triton‐X‐100 for 1 h and blocked with 5% BSA at room temperature for 1 h. Then, these organoids were covered with primary antibody (Table S2) diluted with 1% BSA, and incubated at 4°C overnight. After the first incubation, these organoids were washed three times with ice‐cold PBS containing 0.1% Tween 20 for 5 min each time. Then, the samples were incubated with secondary antibodies for 1 h at room temperature. After washing, the organoids were sealed with glass slides and captured with a spinning disk confocal super‐resolution microscope (Olympus, SpinSR10, Japan).

### 2.10 Live Imaging Observation

We used SIR‐Tubulin (CY‐SC002, Cytoskeleton, USA) to monitor ciliary activity, Hoechst 33258 (C1011, Beyotime, China) to locate the nucleus, MitoTracker (M7514, Sigma) to monitor mitochondrial movement, and BioTracker‐ATP (SCT045, Sigma) was used to monitor ATP location and movement. Then, live images were captured by a spinning disk confocal super‐resolution microscope (Olympus, SpinSR10) after 24 h of staining.

### 2.11 Western Blots

After degumming, organoids were washed with ice‐cold PBS and then lysed with RIPA buffer (Beyotime, P0013C, China) for 1 h on ice. Western blotting was performed as previously described. In brief, protein concentrations were quantified using the BCA protein assay kit (Thermo, 23227, USA) to determine the total protein concentration, isolated protein lysates were run on a 10% SDS‐PAGE gel (Epizyme, PG112, China) and transferred onto PVDF membranes (Millipore, IPVH85R, USA). After blocking with 5% skim milk in TBS‐T at room temperature, PVDF membranes were incubated overnight at 4°C with primary antibody (Table S2). Next, the membranes were incubated with secondary antibodies diluted in a blocking buffer for 1 h at room temperature. The membranes were scanned using a Bio‐Rad ChemiDoc Imaging System or exposed to x‐ray film in a dark room.

### 2.12 Scanning Electron Microscopy Observation

The Matrigel‐containing organoids were washed with ice‐cold PBS, fixed with 2.5% paraformaldehyde supplemented with 2% glutaraldehyde and then stored at 4°C for 2 days. Then, these organoids were divided with a scalpel blade and rinsed three times with PBS (pH 7.4) for 15 min each time. Next, the samples were fixed at room temperature in the dark for 1–2 h with 1% osmic acid and then washed with PBS three times for 15 min each time. Then, the samples were dehydrated in an increasing ethanol gradient (30%, 50%, 75%, 85%, 95% and 100%) and finally immersed in isoamyl acetate for 15 min. The prepared samples were dried in a critical point dryer (Quorum, K850). These samples were placed on conductive carbon film double‐sided tape and placed on an ion sputtering instrument (HITACHI, MC1000, Japan) sample table for gold spraying for ~30 s. Finally, these samples were captured by scanning electron microscopy (TESCAN, VEGA 3 LMU, Czech).

### 2.13 Transmission Electron Microscopy Observation

The Matrigel‐contained organoid was washed with iced PBS, then fixed with 2.5% paraformaldehyde added with 2% glutaraldehyde and then stored at 4°C over 2 days. And then, these organoids were rinsed three times with PBS (pH 7.4), 15 min each time. Next, these samples were fixed in 1% osmic acid at room temperature and kept away from light for 1–2 h before being washed three times with PBS, 15 min each time. And then dehydrated these samples with gradient increased acetone (30%, 50%, 75%, 85%, 95% and 100%). Organoids were embedded with epoxy resin. Using the ultramicrotome (Leica, UC7rt) prepared the ultrathin slices of about 60–90 nm, the slices were spread and then the copper mesh was retrieved. These sections were stained with uranium acetate for 10–15 min and then with lead citrate for 1–2 min at room temperature. Sections were examined with a transmission electron microscope (JEOL, JEM‐1400‐FLASH).

### 2.14 Ciliary Movement Recording

The experiment was captured using an Olympus IX83 high‐speed camera. The total duration of the shot was 5 s at 200 frames per second, which was then imported into ImageJ (1.53K, USA), selected the ‘Line’ tool to draw a line segment perpendicular to the cilia and converted into a waveform image. The waveform diagram was imported into Photoshop (2017CC) software, and after the ruler was converted into pixels, the pixel values of 10 wavelengths were selected and calculated, and the pixel values of a single wavelength were calculated by dividing them by 10. The frequency of motion (Hz) was obtained by dividing the pixel values of a single wavelength by the frame number 200. Then it was put into SPSS 20 for *t*‐value test and variance statistics according to the needs of each group.

### 2.15 ATP Content Detection

This experiment followed the instructions of the ATP Determination Kit (A22066, ThermoFisher). Detailed procedure was listed in Supporting Information.

### 2.16 PRL Content Determination

This experiment followed the instructions of the PRL ELISA Kit (XY9H0259, shxybio). Detailed procedure was listed in Supporting Information.

### 2.17 Human Trophoblast Stem Cell Sphere Attachment Experiment

Based on a 2D co‐culture system [29], we used hTSCs [30] (Human trophoblast stem cells donated by Du Peng Laboratory of Peking University) to mimic embryos to detect whether cilia affect embryo attachment. The first step was to coat the petri dish with collagen IV, and then organoids recovered from Matrigel were spread on the surface until all organoids formed a single‐cell layer. Then, this cell layer was treated with steroid hormones induced secrete stage as a control group, and based on the same treatment, the extra 30 μm ciliobrevin D, a ciliary dynein antagonist was added to inhibit ciliary activity as the experimental group.

The hTSCs were mixed with Matrigel at 10^4^ cells per 1 mL, a 30 μL drop was added to a petri dish, and the cells were covered with culture medium after solidification. After 4 days, these cells formed a sphere in Matrigel. Then, the Matrigel drops were washed with ice‐cold PBS and collected in a 1.5 mL centrifuge tube, put the mixture on ice till Matrigel melted. After centrifugation, the supernatants were discarded, and the spheres were resuspended in a fresh culture medium. Then, using an open‐pulled straw, we selected 20 spheres for the cell layer and measured the adhesion rate after 24 h based on the previous literature [29].

For adhesion rate analysis, we captured the initial figure after 24 h, then used a pipette to gently wash the implanted hTSCs spheres three times with a warmed culture medium and captured the figure again. We divide the number of hTSCs spheres still attached by 20 to get the final adhesion rate.

### 2.18 Statistical Analysis

The data are presented as the means ± standard deviations of at least three independent experiments. The two‐sided Student's *t* test was used to compare two independent groups. The *p* < 0.05 was considered to indicate statistical significance. The statistical analyses were performed using SPSS 20.0 and R 2.10.0.

## 3 Results

### 3.1 Endometrial Epithelial Organoids Establishment and Secretory Phase‐Like State Induction In Vitro

We induced organoids (Figure S1) changed to secretory phase by E2 + P4 + cAMP and samples were collected on Days 10–12 for experiments after hormone stimulation (Figure 1A). As we hypothesized, these epithelial cells showed obvious morphological changes, blank chambers appeared at the cell poles and the cellular height showed a more than twofold increase compared with that of the control group (Figure 1B). By paraffin section H&E staining confirmation of secretory‐stage endometrial tissue from normal people, we found the same volume changes in the luminal and glandular cells which were consistent with the changes in vivo (Figure 1C). ER, PR and PAEP expression changes are established markers for hormone induction in the endometrium, so we assessed these protein expression patterns by immunofluorescence. We found ER+ cell and PR+ cell rates increased after oestrogen stimulation, but decreased after progesterone and cAMP addition (Figure 1D). For PEAP, we observed a positive signal after progesterone stimulation, but not in the control group (Figure 1D). These findings regarding ER [24] and PR [23] expression were consistent with those in previous publications. And luminal liquid detection showed that PRL concentration increased after progesterone treatment (Figure 1E), which participated in endometrial decidualization [23]. Pinopodes, special structures that appear in the early secretory stage, also grew on the surfaces of the luminal membranes of organoids after steroid hormone stimulation (Figures S2 and 3A). Additionally, TEM (Figure S2) showed that the blank chambers were filled with glycogen and that physical changes pushed organelles to the boundary of the cell to achieve a more than twofold cellular height increase (Figure S2). These in vitro findings were similar to those in vivo situations which confirmed the organoids were transformed to the secretory stage upon treatment.

### 3.2 Transcriptomics and Proteomics of the Hormone‐Induced Organoid Model Indicated That Ciliary Growth and IFT‐B Transport Are Critical Processes Involved in the Secretory Phase

**FIGURE 2:**
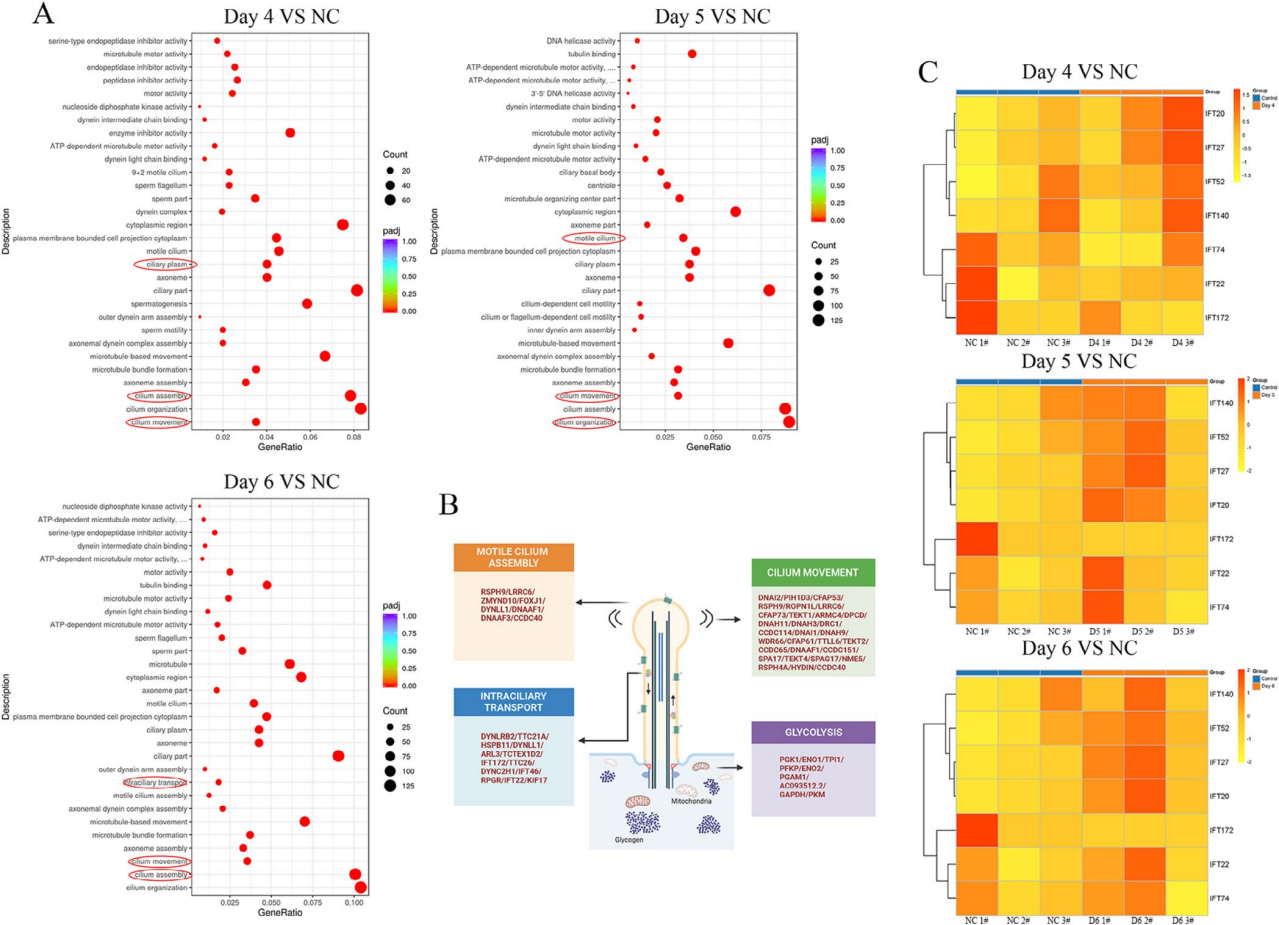
Transcriptomics and proteomics of the hormone‐induced organoid model indicated that ciliary growth and IFT‐B transport are critical processes involved in the secretory phase. (A) GO analyses implied represented by the ciliary assembly, ciliary movement and intraciliary transport, the ciliary activity was a major process during the secretory stage (red frame). (B) Genes detail of significantly increased GO items. (C) Heatmap of dramatically increased intraflagellar transport proteins. Red‐circled terms aims to emphasize these ciliary related processes were upregulated after hormones stimulation.

**FIGURE 3:**
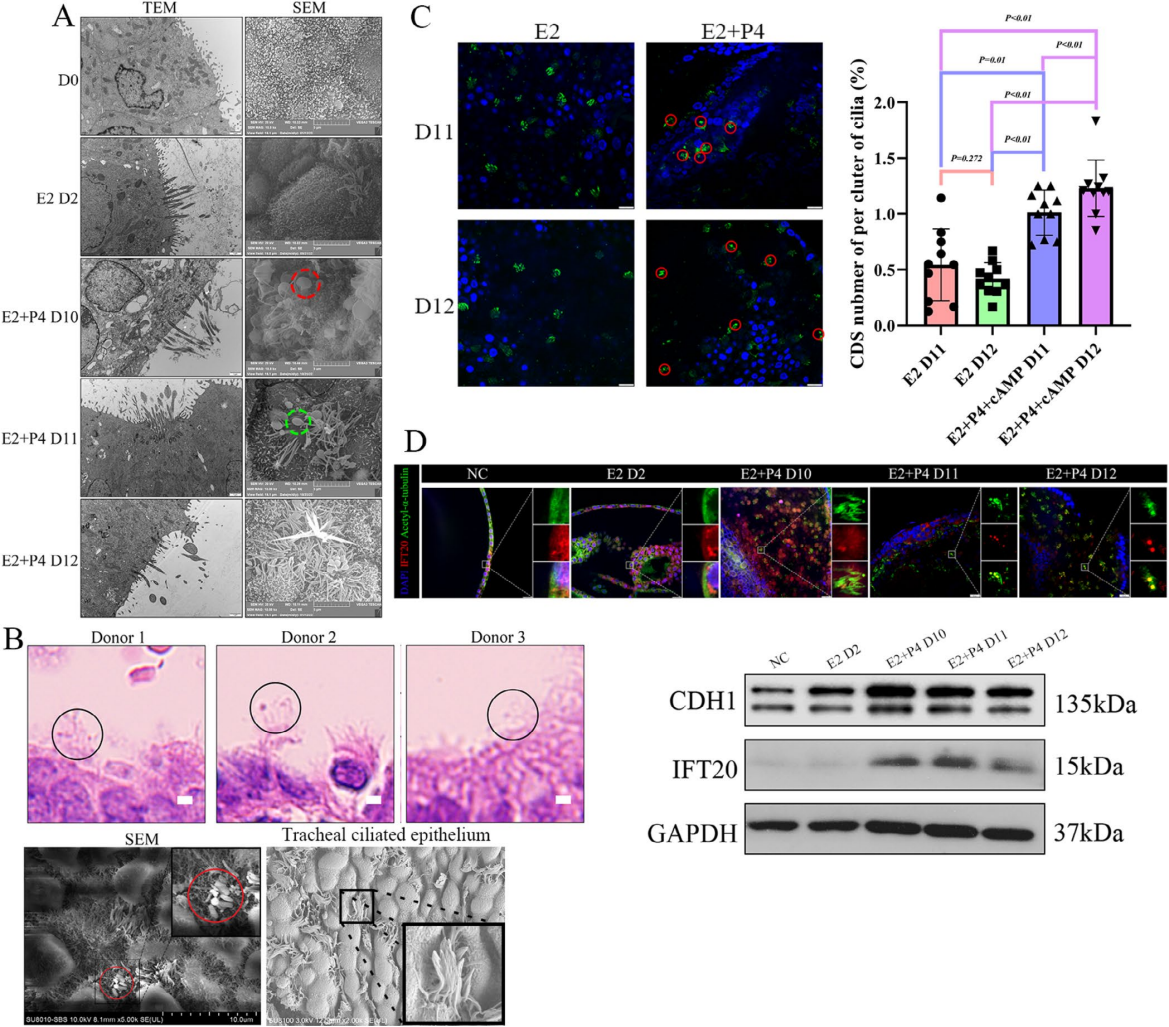
Combined steroid hormone treatment induced dramatic ciliary morphological changes with spheres on the tips, which may function in vesicle transport. (A) Microvilli appeared on the inner surface of organoids but no cilia, ciliogenesis induced by steroids hormones and cilia derived sphere appeared on the apex of cilium after combined hormones stimulation. Scale bars, *TEM*, 1 μm; *SEM*, 5 μm. Red frame: Pinopode; green frame: Cilia‐derived sphere. (B) Cilia‐derived spheres also be found in secretory endometrium in vivo by H&E staining and SEM but not found in other tissues in physiological conditions. Scale bars, *H&E*, 2 μm; *SEM*, 10 μm. (C) Cilia‐derived sphere (red frame) concentrated appearance after progesterone stimulation. Scale bars, 20 μm. a, b, c means *p* < 0.05. (D) IFT20 co‐located with acetylated‐α‐tubulin, concentrated on the CDS after combined hormones stimulation. Partial enlarged detail showed IFT20 located on the chamber of CDS. Scale bars, 20 μm. Its expression level confirmed by WB, increased accompanied with progesterone stimulation. The same trend to CDH1 means many IFT20 participated in endometrial receptivity establishment.

To dissect the molecular changes and these regulatory mechanisms, we performed RNA‐seq analyses of these three organoids of different origins at different stages. Considering that vital changes occurred in the secretory phase, we compared additional progesterone treatment groups (progesterone‐Day 4–6) with the group with no hormones and only added 100% ethanol in the same volume as negative control. PCA showed an obvious bulk cluster difference before and after hormone stimulation (Figure S3A). A total of 134 genes were specifically expressed on progesterone‐Day 4, 357 genes were specifically expressed on progesterone‐Day 5 and 194 genes were specifically expressed on progesterone‐Day 6 (Figure S3B,C). Totally, GO enrich analyses found 111 items in progesterone‐Day 4 versus NC, 87 items in progesterone‐Day 5 versus NC and 141 items in progesterone‐Day 6 versus NC, and items related to cilia were found in groups of all 3 days. Based on *p* value, the most significantly enriched biological processes were related to cilia formation and ciliary movement in each secretory stage compared with the no hormones stimulation control (Figure 2A,B). Furthermore, after pairwise comparison, we found item related ‘focal adhesion’ appeared in progesterone‐Day 5 versus progesterone‐Day 4 (Figure S3D), which may imply that progesterone‐Day 5 is the optimal day of embryo adhesion [21]. Then we performed RT‐qPCR, and our data confirmed these results (Figure S4). Considering the significantly increased ciliary capacity, we performed a proteomics analysis on organoids in the secretory phase to focus on intraflagellar transport (IFT) proteins. Interestingly, increased expression of core proteins of the IFT‐B complex (IFT52, IFT27, IFT20 and IFT22) and IFT140 of the IFT‐A complex was observed (Figure 2C), which were confirmed by immunofluorescence (Figures 3D and S6). Together, the transcriptomic and proteomic results supported that the cilium‐related gene signature was the most significant change after oestrogen combined progesterone induction in the endometrium.

### 3.3 Combined Steroid Hormone Treatment Induced Dramatic Ciliary Morphological Changes With Spheres on the Tips, Which May Function in Vesicle Transport

Prompted by the results of transcriptomic and proteomic sequencing, we further explored the dynamic changes in cilia with both electron microscopy and immunostaining. Scanning electron microscopy (SEM) results showed that canonical pinopodes appeared on the luminal surface of the organoid (Figure 3A). Unexpectedly, we found that an uncanonical structure of cilia appeared on the tops of organoids by electron microscopy, which we named CDSs (Figure 3A). The cilium formed a sphere‐bound axoneme on the apical pole, and these spheres were concentrated on Day 11 and Day 12 after steroid stimulation. These structures were also found in the tissue H&E staining and SEM results from different people at LH + 6 (Figure 3B), implying that these changes existed not only in organoids but also in situ in tissues in the secretory phase. We detected cilia located on the tracheal epithelium (Figure 3B), and checked motile cilia from different tissues published before [31, 32, 33], we found ‘CDS’ is an uncanonical structure that seems typical structure in the secretory stage of endometrium cilia cells.

We hypothesized that IFT along the ciliary axoneme may be the critical factor affecting normal ciliary function. There are no cilia without steroid stimulation (Video S1) but good ciliary growth in the stimulation group (Video S2). Therefore, we attempted to interpret these activity changes in cilia by assessing the formation and function of CDSs. Immunofluorescence showed that the cilia grew well under oestrogen induction alone with fewer ciliary spheres on the top, while they frequently appeared after progesterone addition (Figure 3C). And we found IFT20 co‐localised with acetylated α‐tubulin and was concentrated at the tips of cilia and WB showed a significant increase after stimulation (Figures 3D and S5). CDH1 the protein related to endometrial receptivity establishment [34, 35] was also increased after progesterone treatment. Combined with the transcriptome result, the molecule change of progesterone‐Day 5 may be highly related to embryo adhesion, therefore we also did immunostaining and WB to detect expression of IFT52, IFT27 and IFT22 which belong to IFT‐B core subunits on D11 samples. We found these positive signals on the CDS (Figure S5) and expression levels increased upon hormone treatment (Figure S6). Together, our data support the notion that the IFT‐B complex may participate in ciliary sphere formation after progesterone treatment.

Then, we strived to verify whether these spheres acted as secretory organelles and affected ciliary movement. By live imaging, we noticed that there were numerous vesicles located on the sphere (Video S3). Together, our results support the idea that hormone stimulation enhances IFT in cilia and enriches vesicles in CDSs.

### 3.4 Microdissection Combined Ultra‐Microproteome Verified Metabolic Enriched Protein Components Reflecting the Role of Endometrial Epithelial Cilia

**FIGURE 4:**
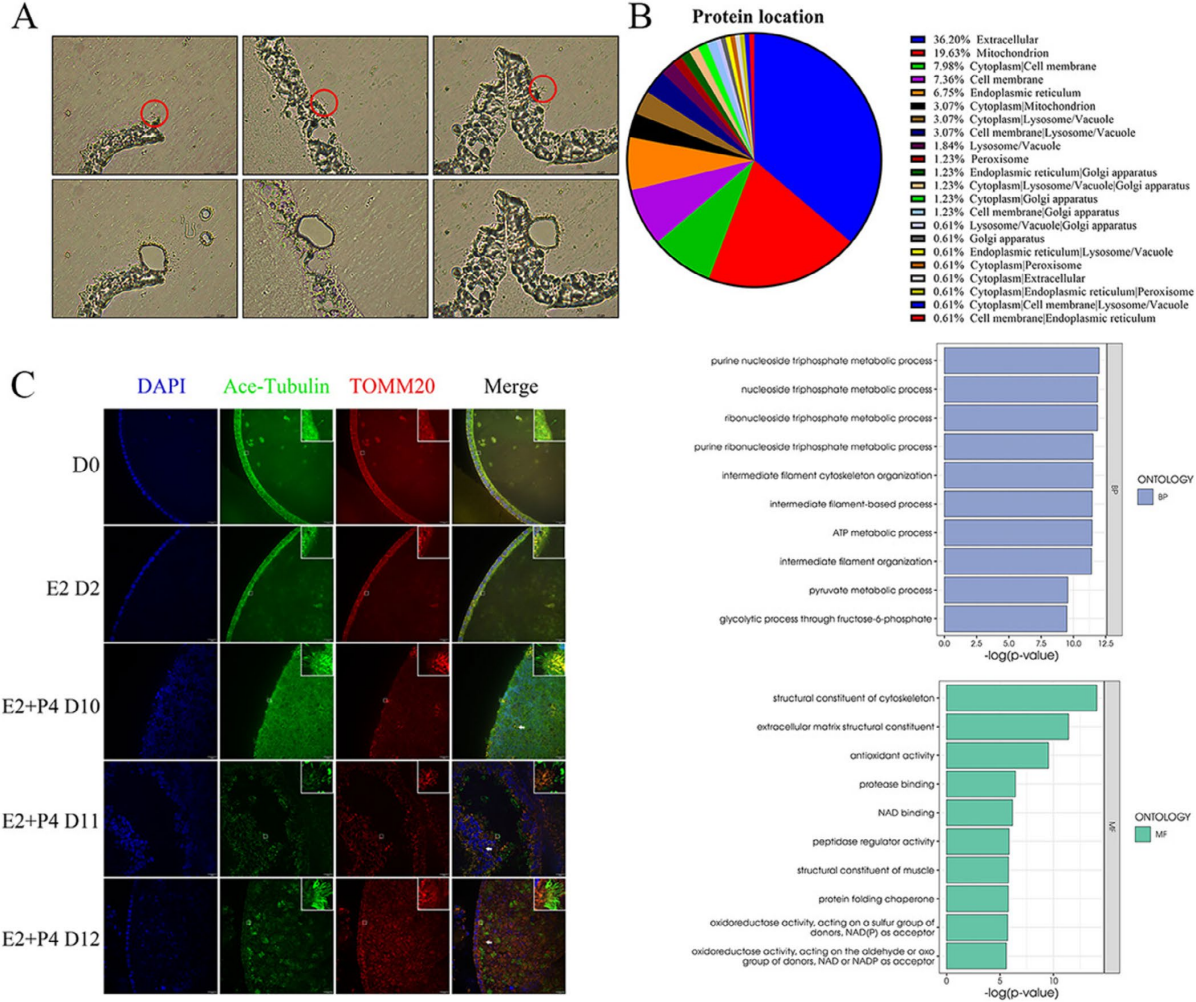
Microdissection combined ultramicroproteome verified protein components of endometrial epithelial cilia may participate in endometrium microenvironment establishment. (A) Totally, 650 cilia spheres were collected. Scale bars, 15 μm. (B) Protein components of endometrial epithelial cilia. (C) TOMM20 represented mitochondria from dispersed distribution transformed to concentrated at the bottom of cilia. Part of these mitochondria formed like a donut. Scale bars, 20 μm. The experiment was repeated three times.

As mentioned above, hormones stimulated organoids induced cilia formation with dramatically increased mitochondrial and energy metabolic activity (Figure 2B). We were then curious about the protein components as these proteins may be secret from cilia to the luminal environment to affect embryo implantation. As shown in Figure 4, by microdissection, we separated cilia containing CDSs to analyse the protein contents. Ultramicroproteome results showed that 481 proteins were detected, most of them enriched in extracellular, the second is mitochondria, part of them belongs to endoplasm reticulum, lysosome and other cellular organelles. Biological process analyses showed that ATP and pyruvate metabolic activity were enriched in cilia. We also found some proteins such as: RPLP0, RPLP1, RPLP4, RPLP8, RPLP10 and RPLP13, belong to ribosomal subunits, this finding was in agreement with the recent study indicating a local translation along cilia structure [36]. Therefore, to sustain high synthesis activity, the increased local energy metabolic activity was needed (Figure 4A). For GO analyses, molecular function and biological process showed a high relationship to energy metabolism and cilia formation. We also found secretory protein like S100A9 which is in accordance with the established role in embryo implantation located on cilia confirming that it performs secretory function [37].

We hypothesize mitochondrial proteins may function to support ciliary local synthesis. By immunofluorescence staining of the organoid which was stimulated by combined hormones for 5 days, we found mitochondria (Figure 4B and Video S4) appeared polarised distribution enriched at the bottom of cilia, and signal of TOMM20 confirmed that mitochondria located at the bottom of cilia (Figure 4C). Together, these analyses implied dramatically increased activity in protein synthesis to sustain cilia formation to ensure protein transportation and secretion to support embryo implantation.

### 3.5 Defective Ciliary Intraflagellar Transport and Mitochondria Function Were Related to Compromised Implantation Potential in Endometrial Organoids From RIF Patients

**FIGURE 5:**
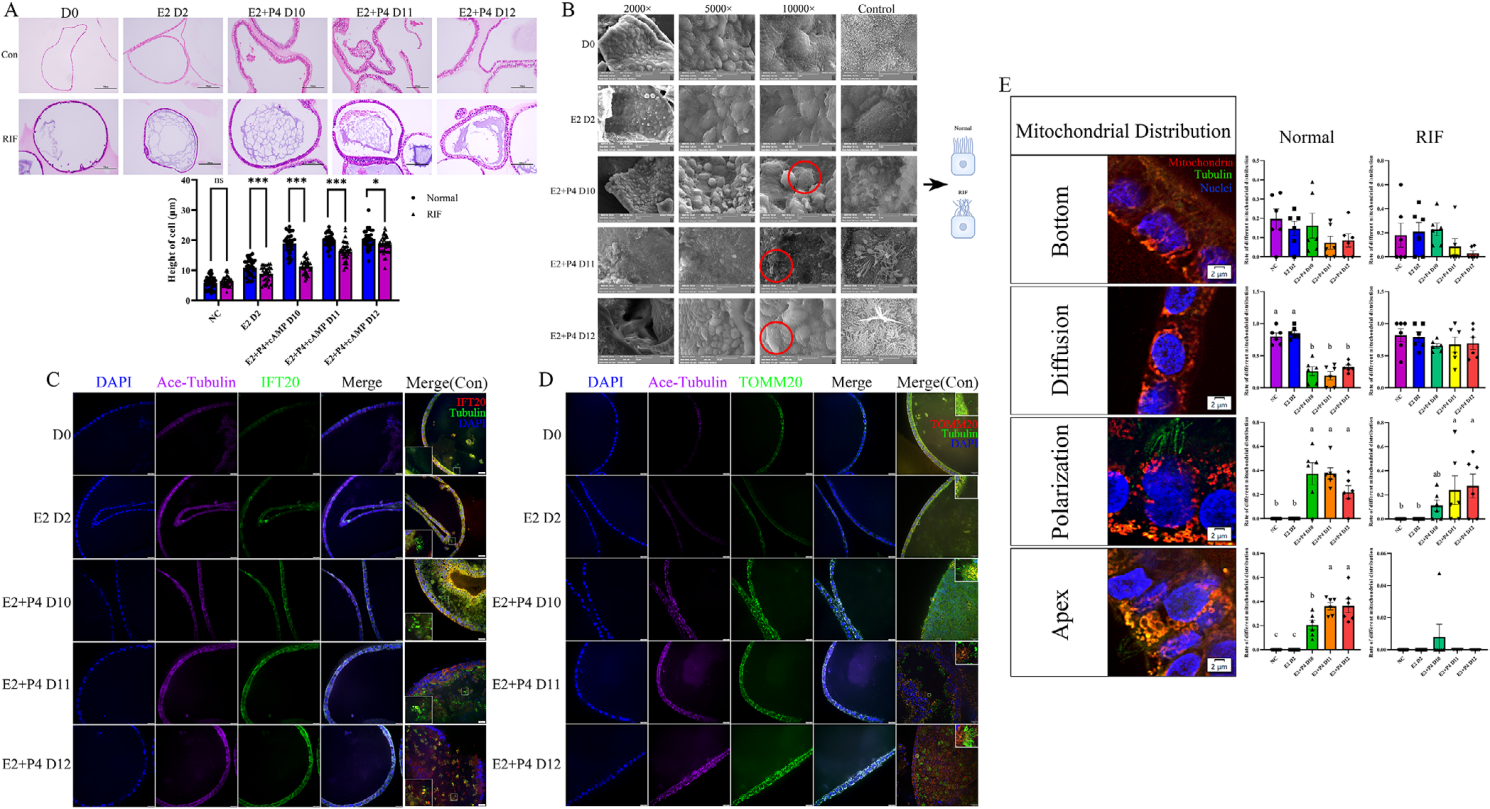
Defective ciliary intraflagellar transport and mitochondrial distribution were found in endometrial organoids from RIF patients after combined hormones treatment. (A) There was no significant change in cell volume in RIF organoids. Scale bars, 100 μm. (B) SEM results detected cilia on the inner surface of RIF organoids were bundled together. Scale bars, *Left*, 20 μm; *Middle*, 10 μm; *Right*, 5 μm. (C) Lack of ciliary acetylation led to defective intraflagellar transport. Scale bars, 20 μm. The experiment was repeated three times. (D) TOMM20 presented dispersed distribution no longer polar distribution. Scale bars, 20 μm. The experiment was repeated three times. (E) The four different patterns of mitochondrial distribution in endometrial epithelial cells in normal and RIF groups. The right panel is the statistics of four pattern rates in two groups at different hormone stimulation stages. a, b, c means *p* < 0.05 with a significant difference.

**FIGURE 6:**
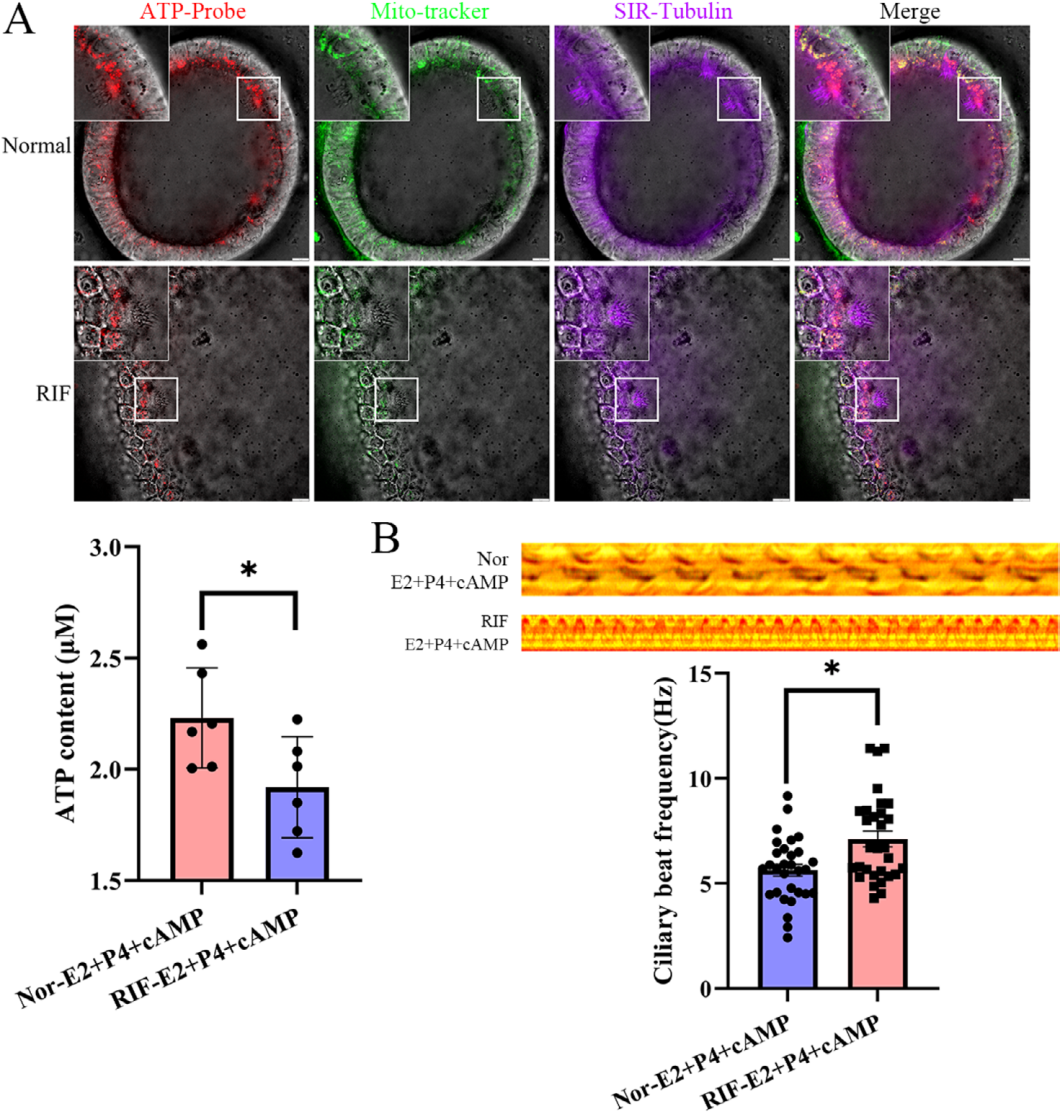
Ciliary movement of RIF faster than normal even though the lower ATP content. (A) Live imaging probe shows ATP and mitochondria co‐location and concentration at the bottom of cilia. ATP determination showed lower ATP content of RIF compare to normal. Scale bar, 20 μm. *p* < 0.05. (B) Cilia movement oscillograph. *p < 0.05.

All of these findings in normal organoids showed that ciliogenesis and morphological changes were accompanied by receptive status establishment, which indicates that there is a relationship between cilia and embryo attachment. We speculated that RIF featured defective ciliogenesis and related metabolic dysfunction after hormone stimulation. H&E staining showed that the volume changes could not reach the scale we found in normal samples (Figure 5A). We found that the cilia located on the surface of RIF organoids were stuck together as shown by SEM (Figure 5B). Unexpectedly, we found a positive acetylated α‐tubulin signal on the cytoskeleton except for cilia in organoids from RIF patients (Figure S7), and our results showed that IFT‐20 and other subunits of IFT‐B were located in the cytoplasm but had weak signals on the cilia (Figures 5C and S7). These subunits of IFT‐B did not concentrate at the tips of the cilia to form a sphere. In accordance with our finding on mitochondria, we noticed that the TOMM20 signal in the RIF group shows diffused in the cytoplasm, and it no longer had the characteristic polar distribution (Figure 5D). Furthermore, to understand the possible relationship between mitochondrial dynamics with ciliogenesis [38, 39, 40], we captured four typical figures in different stages to define four patterns of mitochondria distribution: bottom, diffusion, polarisation and apex. We found mitochondria will concentrate at the bottom of cilia showing ‘Polarisation’ and ‘Apex’ two patterns, and showed ‘diffused’ and ‘bottom’ without cilia. Also the mitochondria located on the bottom showed no difference whatever hormone stimulation, but polarisation and apex pattern showed a significant increase after hormone treatment (Figure 5E) which was not shown in the RIF grouInterestingly, mtDNA level detection showed that mitochondria DNA copy number was increased after hormone stimulation in RIF samples (Figure S8), and it is a possible compensational mitochondrial biogenesis increase for energy supplement deficiency. Furthermore, we found ATP enriched at the bottom of cilia co‐located with mitochondria in normal organoids while less signal was detected in RIF, and these results were confirmed by ATP determination (Figure 6A). To our surprise, ciliary movement in RIF was higher than normal (Figure 6B), and the underlying cause of excessive activity is related to, over‐expenditure of energy and inability to perform implantation‐related biological activities, which result in lower ATP and glycogen content with excessive ciliary movement induced embryo adhesion failure [14, 15]. In conclusion, both cilia‐related IFT transport and the mitochondrial dynamics were obviously deficient in RIF organoids.

Finally, we used an implantation model to verify whether ciliary activity affects the implantation process. Organoids were degummed, set in Petri dishes coated with collagen I (C7661, Sigma) and then treated with culture medium containing 30 μM ciliobrevin D (S9743, Selleck) the cilia dynein antagonist and combined hormones for 4 days when they became flattened. Then, we set 20 hTSCs balls on the surface of the cell layer and assessed them after 24 h. Unlike the hormone stimulation group, the ciliobrevin D‐treated group had fewer hTSCs balls attachment and we found that ciliary structure protein acetylated α‐tubulin expression is significantly decreased (Figure S9); while more than 10 balls adhered to the cell layer in the hormone group (Figure S10). These results implied that integrated cilia transport was necessary for IFT and may be needed for embryo implantation.

## 4 Discussion

In the current study, detailed molecular characterisation and time‐lapse analyses of organoids derived from normal people and RIF patients via hormone induction in vitro revealed concrete evidence showing the critical roles of cilia during human endometrial receptivity establishment. For the first time, our current study reveals novel CDS structures that may participate in metabolic regulation during secretory endometrium‐receptive microenvironment establishment. Furthermore, our results support the idea that mitochondria‐related energy metabolism coordinates the regulation of the endometrial microenvironment by motile cilia. Steroid hormones induce endometrial ciliogenesis and form a sphere after progesterone addition, possibly as a result of activated IFT. Several key points indicated that mitochondria‐related processes were critical for the above‐mentioned ciliary transport, which was compromised in endometrial epithelial cells from RIF patients. First, immunofluorescence revealed that subunits of the IFT‐B complex were concentrated on the spheres in normal organoids but not in RIF organoids. Meanwhile, mitochondria migrated to the base of the cilium to accelerate IFT via energy supply, yet mitochondria of RIF presented a diffuse distribution with an increase in the DNA copy number. Furthermore, we found an absence of acetylated α‐tubulin in RIF organoids, and integrated cilia were necessary for IFT and may have participated in embryo implantation, as shown by the in vitro implantation experiment. All of these results suggested that mitochondria‐accelerated motile cilia IFT may be involved in endometrial microenvironment establishment (Figure S11).

Accumulating data have shown that cilia formation and degeneration are dynamically regulated, especially at the window of implantation, and that pinopodes cover the cilia [41]; therefore, the behaviour of cilia is often ignored. With the limitations of previous culture methods and the lack of an appropriate in vitro model, we can only choose a certain point in time to assess the functions of cilia in implantation without dynamic observation. Therefore, organoids provide an excellent platform to study the changes and functions of cilia during implantation. Motile cilia are usually present on the surfaces of epithelial cells [42]. The IFT complex is composed of IFT‐B and IFT‐A; these two complexes are responsible for anterograde and retrograde transport [43]. In this study, we found that the expression of the IFT‐B complex subunits IFT52, IFT22, IFT27 and IFT20 was increased, indicating significantly increased transport activity from the basement to the apex. Furthermore, cargos transported by IFT‐B were enriched at the apex so that the apical cilia formed a sphere. In fact, IFT transport has been shown to be critical for protein transport and ciliogenesis. Research on *Trypanosoma brucei* has proven that IFT22 is essential for flagellum assembly [44], and another article has confirmed that the IFT22/RABL5 complex recruits the BBsome to the basal body, participating in ciliary transport [45]. IFT52 is a core protein of IFT‐B1b that interacts with IFT88 [46], which plays a vital role in cilium assembly [47], and its mutation can induce cilium disruption and result in short rib polydactyly syndrome [48]. IFT20 constitutes the part of the peripheral complex of IFT‐B that interacts with IFT38, IFT57 and IFT54 [46], which is responsible for many important metabolic processes and signal transduction. All these findings imply that ciliogenesis is highly related to the IFT‐B complex and that cilia may play an important role in the implantation process.

The detailed mechanism of how the dynamic location changes mitochondrial protein to ciliary function needs further clarification. Interestingly, we found that donut‐shaped mitochondria seem to play important roles in ciliogenesis, IFT transport and implantation. It has been shown that donut‐shaped mitochondria are highly enriched in cells with highly glycolytic muscle fibres, while other forms of mitochondria are more common in oxidative muscle fibres [49]. And also, donut‐shaped mitochondria formation increased highly related to mitophagy during osteoblast maturation and enhanced mitochondrial secretion to promote osteogenesis [50]. On the other hand, donut‐shaped mitochondria may play a message from mother to baby to activate AMPK and regulate DBL‐1/TGFβ to control growth and metabolism to increase the offspring adult body size [51]. Therefore, it is possible that the donut‐shaped mitochondria of the current model accommodate the high‐glucose environment during implantation window establishment and that their function, at least partially, is to provide metabolic flexibility for ciliogenesis and IFT transport [52]. Another pressing question that needs to be answered is how the CDS structure in the ciliary tip is formed in the endometrium during the implantation window. Although rarely reported, a few studies on a BBsome‐subunit‐KO mouse model have shown that tracheal cilia from BBS4‐KO mice develop a structure that somehow mimics the CDS structure shown in our model [53]. Research on the BBsome has shown that it can regulate mitochondrial dynamics and function in the brain and that BBS8 deficiency can induce mitochondrial hyperfusion [54]. Notably, BBsome recruitment is regulated by the IFT22/RABL5 complex. Consistent with these findings, in our study we found that IFT22 expression was significantly increased and that mitochondria were concentrated at the bases of cilia. Nevertheless, how these BBsome proteins and mitochondrial machinery coordinate CDS formation and whether the structure is involved in endometrial receptivity establishment warrant further clarification.

## 5 Conclusion

In this study, we used endometrial organoids together with patient samples and identified a novel morphology of endometrial epithelial cilia after hormone stimulation, which may be the result of IFT through mitochondrial dynamic changes. The emerging complex IFT‐B is involved in ciliogenesis, and related novel CDS structures may participate in metabolic regulation during secretory endometrium receptive microenvironment establishment.

## Author Contributions

Haoxuan Yang conducted the experiments, interpreted the results and drafted the manuscript. Jing Zhang conducted the experiments and drafted the manuscript. Fei Yan and Yihong Chen performed the literature search and drafted the figures. Yang Wu, Lian Duan, Juan Zou, Jiuzhi Zeng, Weixin Liu and Xue Xiao collected the samples. Juncen Guo and Jiyun Pang drafted the figures. Andras Dinnyes, Chi Chiu Wang and Yi Lin edited the manuscript. Xiaomiao Zhao and Xue Xiao interpreted the results and edited the manuscript. Wenming Xu conceptualised the study and edited the manuscript.

## Conflicts of Interest

The authors declare no conflicts of interest.

## Supporting information


**Figure S1.** Endometrium‐derived organoids establishment. (A) Organoids develop after digestion. Scale bars, 200 μm. (B) Different passage of organoids derived from three donors. Scale bars, 200 μm. (C) Immunostaining of organoids for endometrial epithelial cell markers (CK18, MUC1, CDH1 and LIF) checked cell type and endometrial characteristics. Scale bars, 200 μm. The experiment was repeated three times.

**Figure S2.** Organoids transformed to a secretory phase‐like state after steroid hormone stimulation. (A) Pinopodes appeared on the surface of organoids in the E2 + P4 condition. P indicates pinopode; the black arrow indicates mitochondria; the white arrow indicates vesicle; the red arrow indicates endoplasmic reticulum and the yellow arrow indicates microvilli. Scale bars, 2 μm. (B) Cell volume changes induced by progesterone. P indicates pinopode; the black arrow indicates cilia; the white arrow indicates glycogen and the red arrow indicates mitochondria. Scale bars, *upper*, 2 μm; *middle*, 2 μm; *bottom first*, *second*, 5 μm; *bottom last*, 2 μm. The experiment was repeated three times.

**Figure S3.** Details of RNA‐sequencing. (A) PCA analysis showed an obvious bulk cluster difference between before and after hormone stimulation. (B) These groups all expressed 11,783 genes and other 518 genes were expressed on all three secretory stages, 373 genes were specifically expressed in the control group, 134 genes were specifically expressed on progesterone‐Day 4, 357 genes specifically expressed on progesterone‐Day 5 and 194 genes were specifically expressed on progesterone‐Day 6. (C) Volcano plot of RNA‐sequencing. Progesterone‐Day 4/5/6 means the days after combined hormones stimulation. (D) GO analysis results implied progesterone‐Day 5 be may the day for embryo implantation. The red frame emphasised ‘focal adhesion’ which was related to embryo implantation.

**Figure S4.** Genes confirmation for RNA‐sequencing.

**Figure S5.** IFT‐B subunit expression increased after hormone stimulation.

**Figure S6.** IFT complex concentrated on the CDS. IFT22, 52 and 27 signals were co‐located with acetylated‐α‐tubulin and were concentrated on the CDS after combined hormones stimulation. These most increased subunits represented IFT complex concentrated on the CDS implied increased activity of intraflagellar transport after steroid hormones stimulation. Scale bars, 20 μm. The experiment was repeated three times.

**Figure S7.** IFT complex of RIF organoid disappeared at the CDS at D11 and D12. Because of the lack of ciliary acetylation, these subunits of the IFT complex are only enriched at the cell submembrane but no longer concentrated on the CDS. Scale bars, 20 μm. The experiment was repeated three times.

**Figure S8.** RIF organoid showed an increased number of mitochondrial DNA copy. *p* < 0.05.

**Figure S9.** Ciliobrevin D induced ciliary injury. Cilia‐specific antagonist ciliobrevin D treatment induced ciliary acetylated microtubule deficiency so that their length was shorter than normal. Scale bars, 20 μm. *p* < 0.01.

**Figure S10.** The hTSCs ball cannot adhere to the epithelial cell after anti‐cilia reagent treatment. We set 20 hTSCs balls on the surface of the cell layer, and check it after 24 h. Compared with more than 10 balls firmly adhesion in the normal group, hTSCs balls got loose adhesion in the ciliobrevin D treated and RIF group and easily washed away by pipette. After washing, the rest balls number divide by 20 to get the final adhesion rate. Scale bars, 100 μm. *p* < 0.05.

**Figure S11.** Mitochondria‐accelerated motile cilia intraflagellar transport supplied by glycogenesis and glycolysis energy may be involved in endometrial microenvironment establishment.

**Table S1.** Primer list for RT‐qPCR and digital PCR.

**Table S2.** Antibodies and live imaging probe list for WB and immunofluorescence.

**Video S1.** The 3D layer scanning observation of endometrial organoids showed no cilia appeared on the inner surface (organoids stained with Hoechst, SIR‐Tubulin and Mito‐Tracker, nuclei showed blue, tubulin showed red and mitochondria showed green). Scale bars, 10 μm.

**Video S2.** The 3D layer scanning observation of endometrial organoids showed cilia appeared on the inner surface after combined hormones stimulation (organoids stained with Hoechst, SIR‐Tubulin and Mito‐Tracker, nuclei showed blue, tubulin showed red and mitochondria showed green). Scale bars, 10 μm.

**Video S3.** Cilia sphere containing vesicles in the hollow centre (red circle indicates cilia sphere). Video recorded by super‐resolution microscope. Scale bars, 10 μm.

**Video S4.** Mitochondria enriched at the bottom of cilia (organoids stained with Hoechst, SIR‐Tubulin and Mito‐Tracker, nuclei are blue, tubulin is red and mitochondria are green). Imaging by time‐lapse observation over 10 min with images captured every 30 s. Scale bars, *Full* 20 μm; *Video in frame*, 2 μm.

Data S1.

## Data Availability

The data that support the findings of this study are available from the corresponding author upon reasonable request.
