# Supplementary figures and images for: Ciliary IFT‐B Transportation Plays an Important Role in Human Endometrial Receptivity Establishment and is Disrupted in Recurrent Implantation Failure Patients
Source: Cell Prolif. 2025 Feb 6;58(7):e13819. doi: 10.1111/cpr.13819 (PMC12240636; doi:10.1111/cpr.13819)

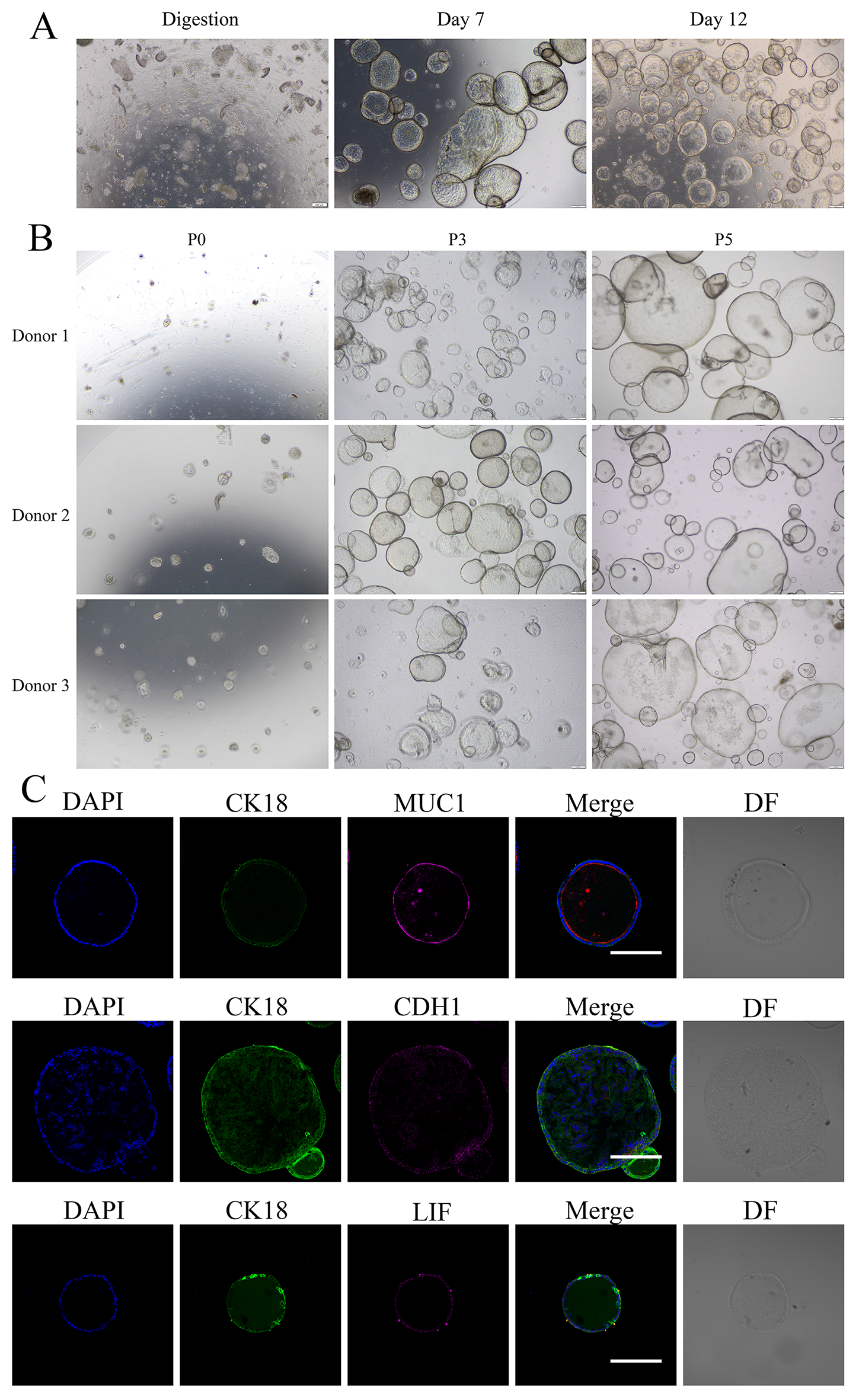

Supplement: Supplementary file 1 — Figure S1. Endometrium‐derived organoids establishment. (A) Organoids develop after digestion. Scale bars, 200 μm. (B) Different passage of organoids derived from three donors. Scale bars, 200 μm. (C) Immunostaining of organoids for endometrial epithelial cell markers (CK18, MUC1, CDH1 and LIF) checked cell type and endometrial characteristics. Scale bars, 200 μm. The experiment was repeated three times. [file CPR-58-e13819-s008.tif]

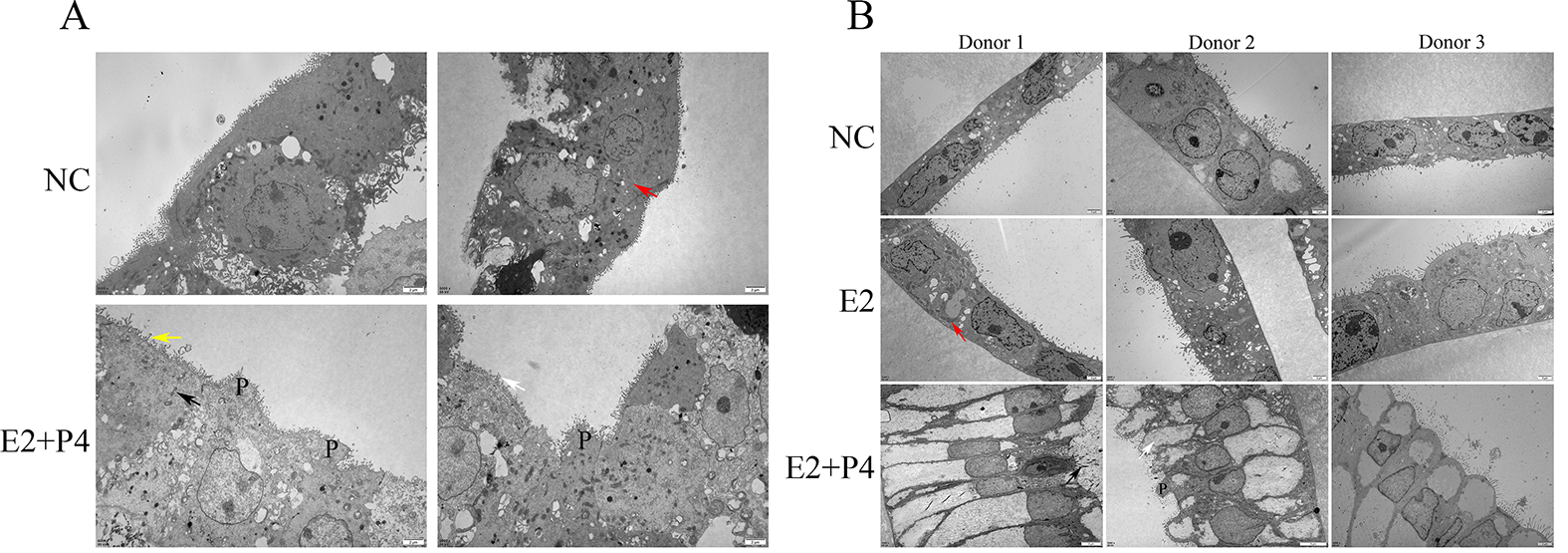

Supplement: Supplementary file 2 — Figure S2. Organoids transformed to a secretory phase‐like state after steroid hormone stimulation. (A) Pinopodes appeared on the surface of organoids in the E2 + P4 condition. P indicates pinopode; the black arrow indicates mitochondria; the white arrow indicates vesicle; the red arrow indicates endoplasmic reticulum and the yellow arrow indicates microvilli. Scale bars, 2 μm. (B) Cell volume changes induced by progesterone. P indicates pinopode; the black arrow indicates cilia; the white arrow indicates glycogen and the red arrow indicates mitochondria. Scale bars, upper, 2 μm; middle, 2 μm; bottom first, second, 5 μm; bottom last, 2 μm. The experiment was repeated three times. [file CPR-58-e13819-s010.tif]

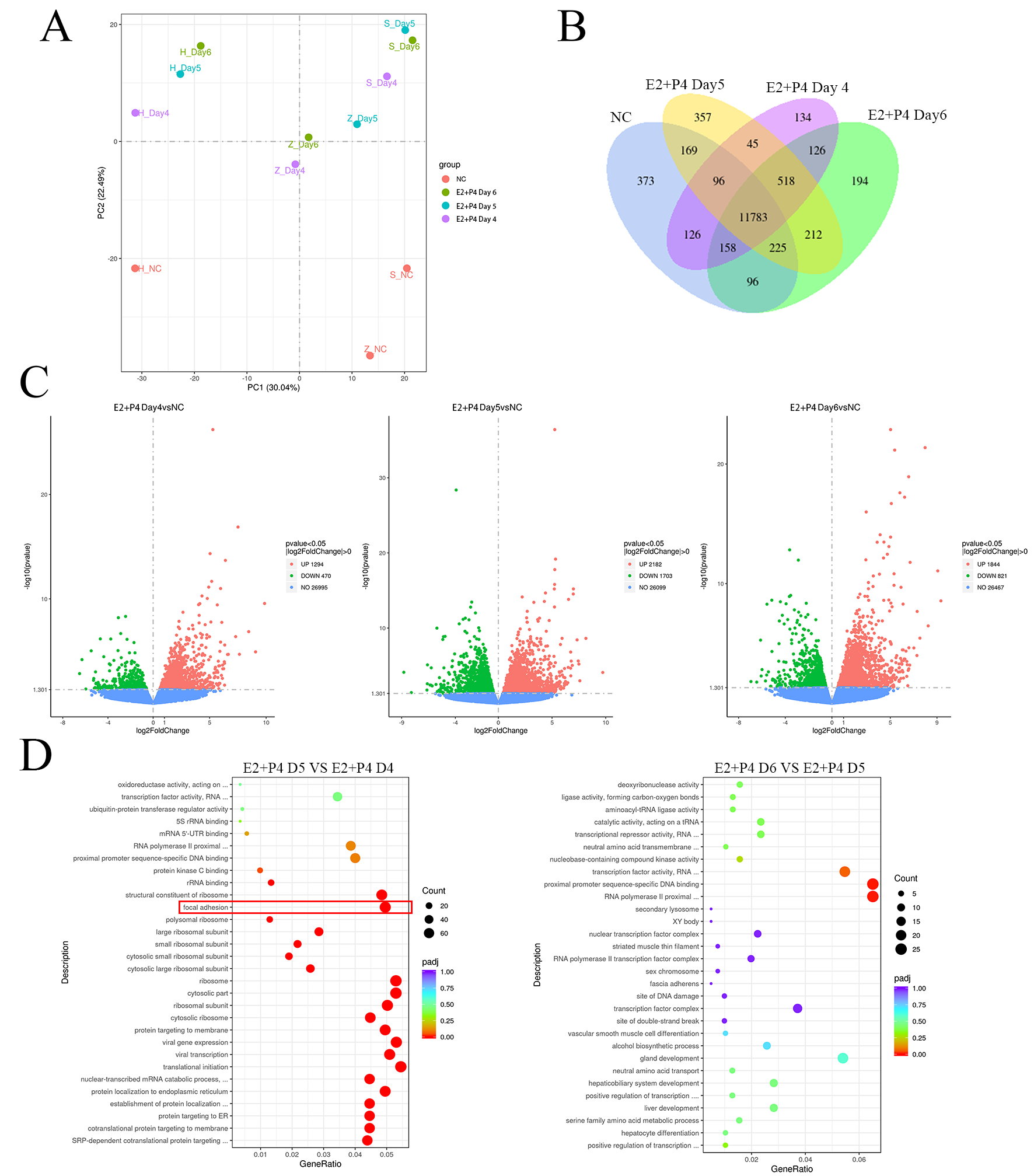

Supplement: Supplementary file 3 — Figure S3. Details of RNA‐sequencing. (A) PCA analysis showed an obvious bulk cluster difference between before and after hormone stimulation. (B) These groups all expressed 11,783 genes and other 518 genes were expressed on all three secretory stages, 373 genes were specifically expressed in the control group, 134 genes were specifically expressed on progesterone‐Day 4, 357 genes specifically expressed on progesterone‐Day 5 and 194 genes were specifically expressed on progesterone‐Day 6. (C) Volcano plot of RNA‐sequencing. Progesterone‐Day 4/5/6 means the days after combined hormones stimulation. (D) GO analysis results implied progesterone‐Day 5 be may the day for embryo implantation. The red frame emphasised ‘focal adhesion’ which was related to embryo implantation. [file CPR-58-e13819-s003.tif]

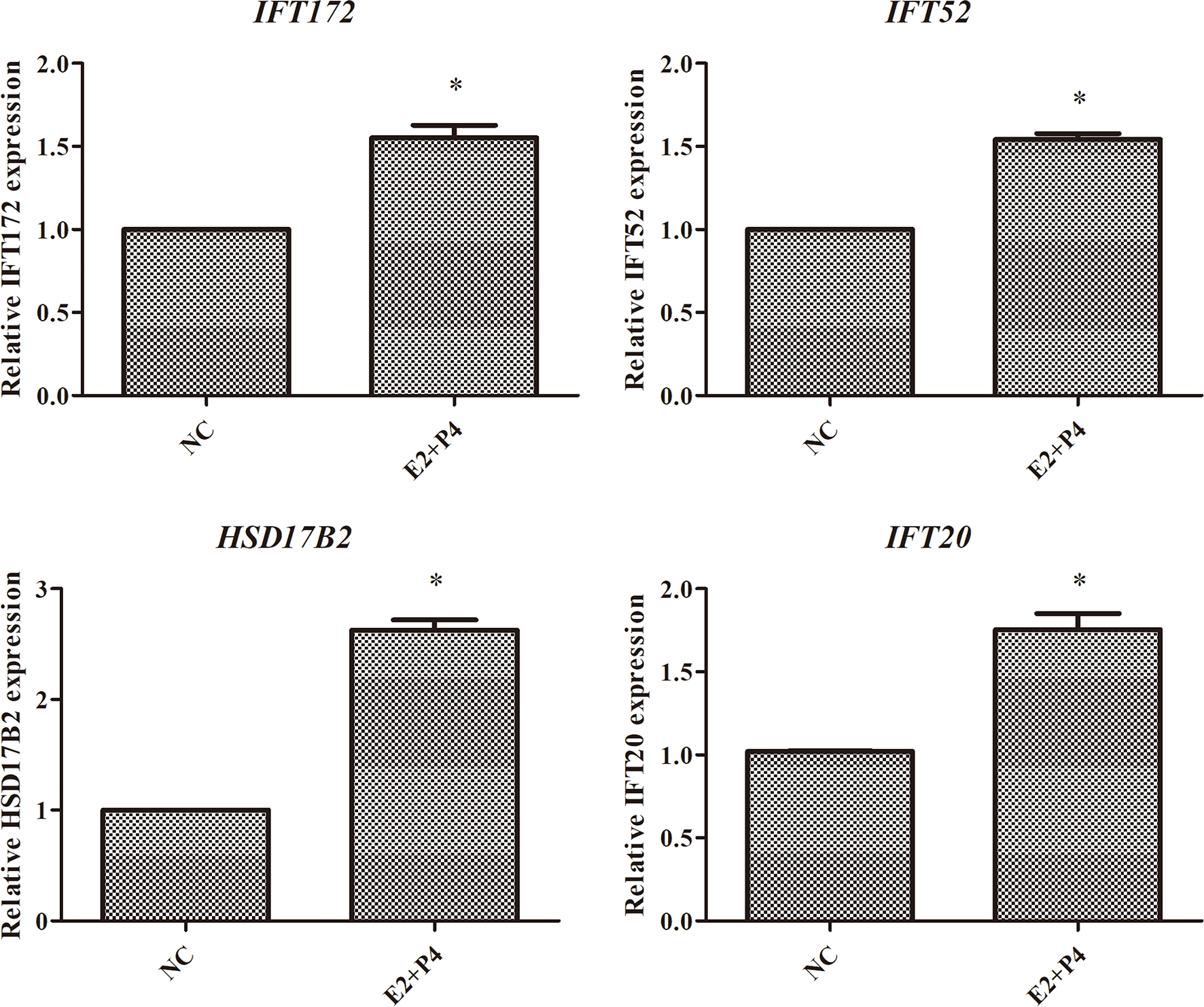

Supplement: Supplementary file 4 — Figure S4. Genes confirmation for RNA‐sequencing. [file CPR-58-e13819-s005.tif]

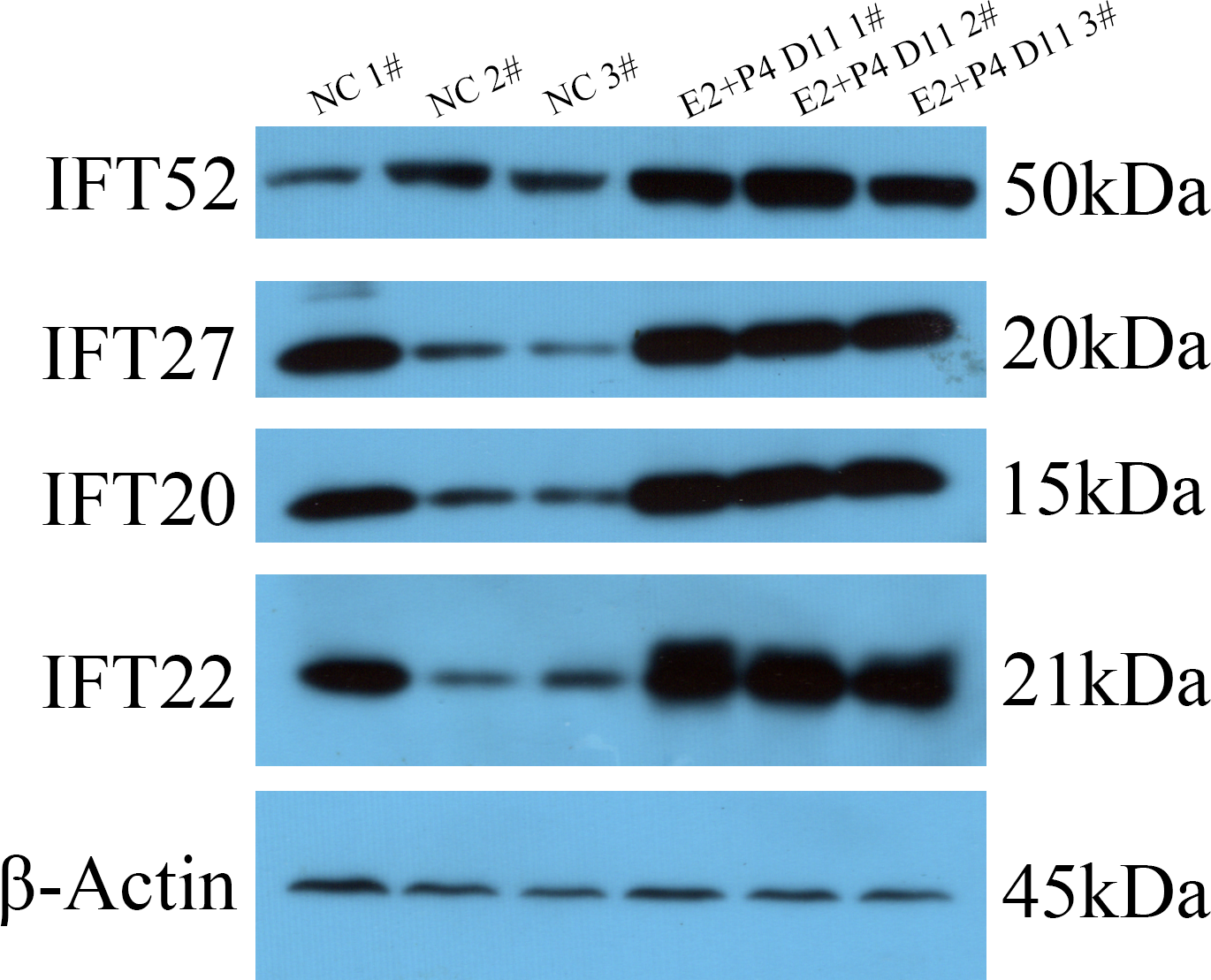

Supplement: Supplementary file 5 — Figure S5. IFT‐B subunit expression increased after hormone stimulation. [file CPR-58-e13819-s015.tif]

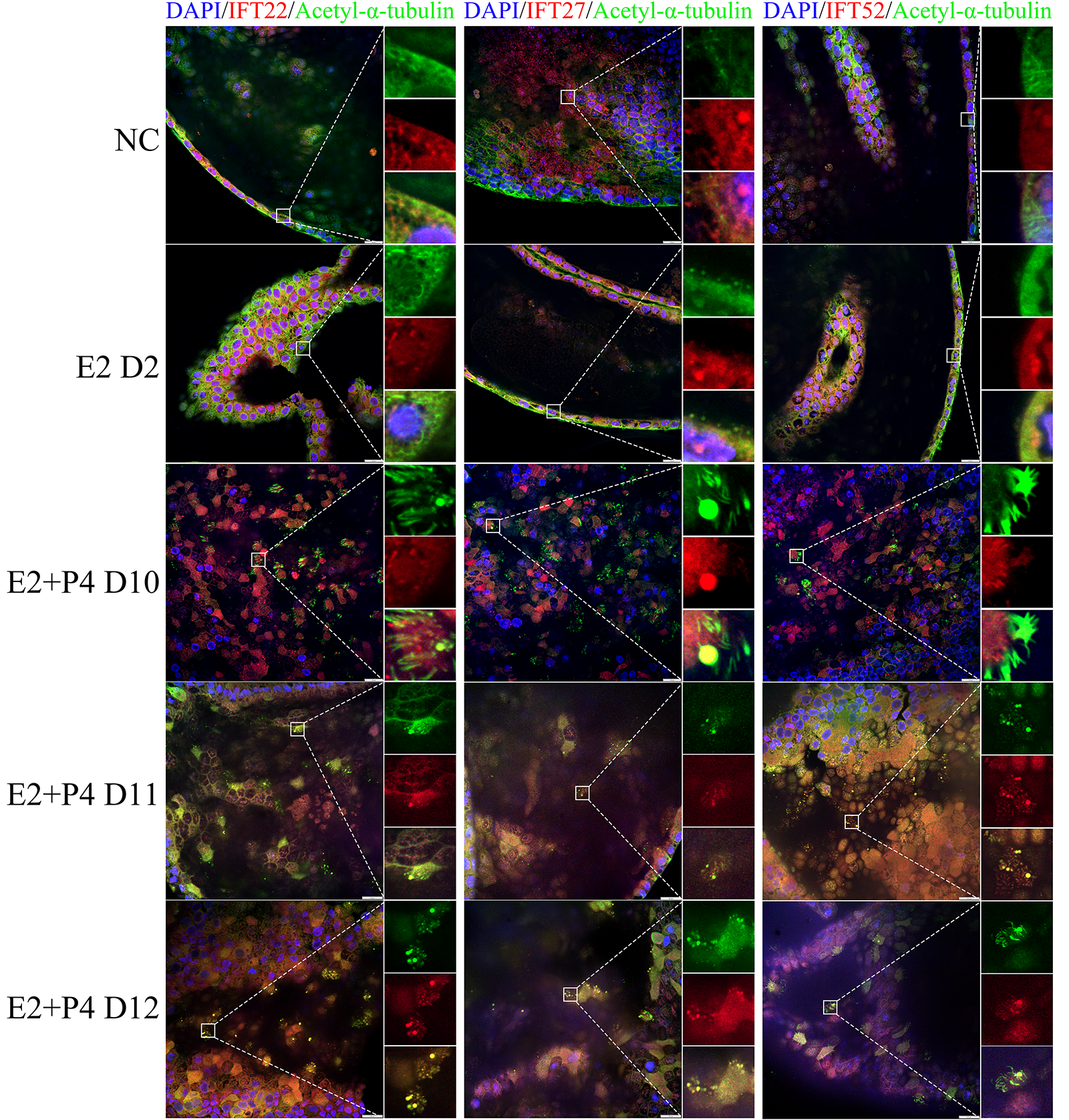

Supplement: Supplementary file 6 — Figure S6. IFT complex concentrated on the CDS. IFT22, 52 and 27 signals were co‐located with acetylated‐α‐tubulin and were concentrated on the CDS after combined hormones stimulation. These most increased subunits represented IFT complex concentrated on the CDS implied increased activity of intraflagellar transport after steroid hormones stimulation. Scale bars, 20 μm. The experiment was repeated three times. [file CPR-58-e13819-s017.tif]

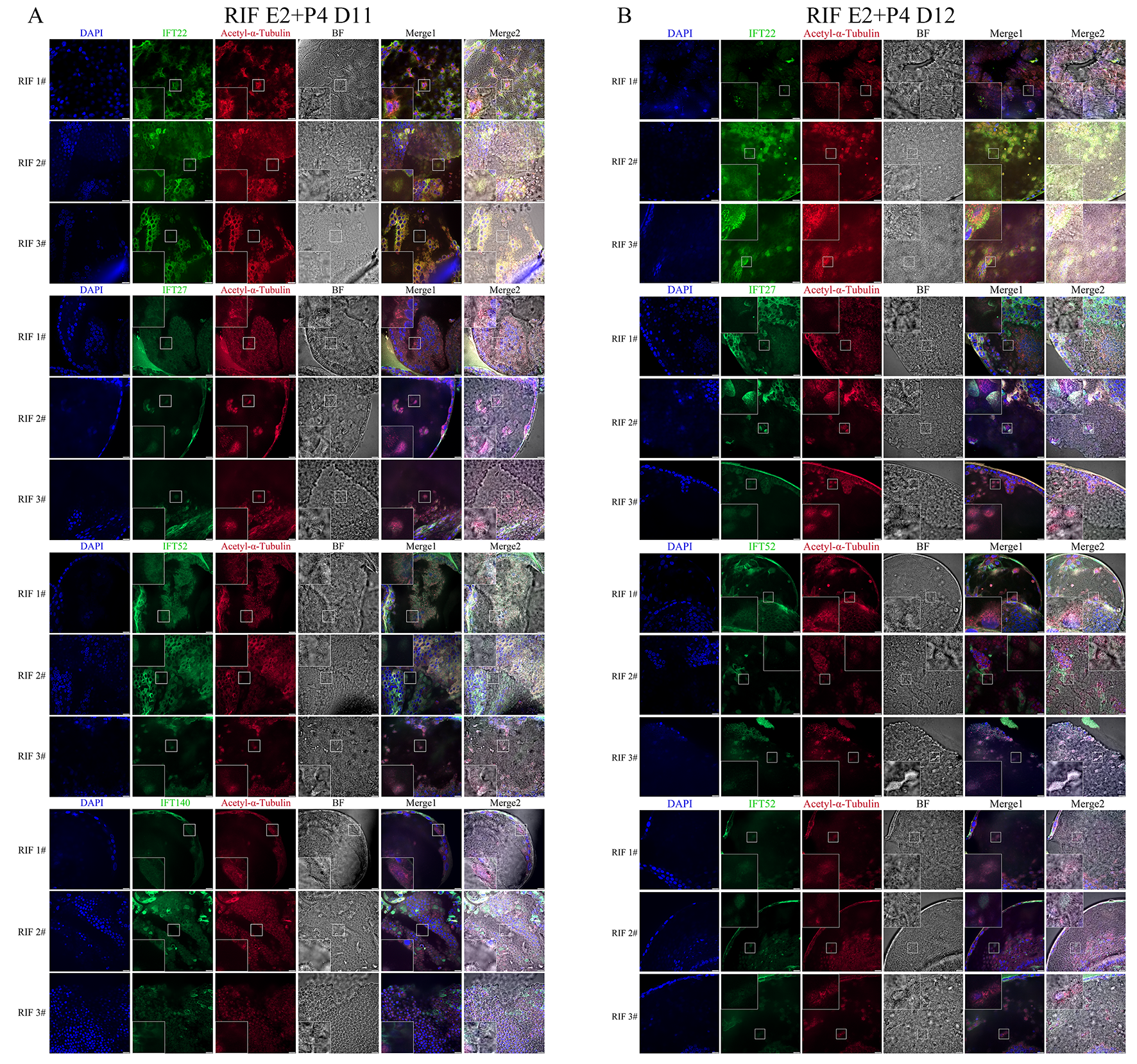

Supplement: Supplementary file 7 — Figure S7. IFT complex of RIF organoid disappeared at the CDS at D11 and D12. Because of the lack of ciliary acetylation, these subunits of the IFT complex are only enriched at the cell submembrane but no longer concentrated on the CDS. Scale bars, 20 μm. The experiment was repeated three times. [file CPR-58-e13819-s007.tif]

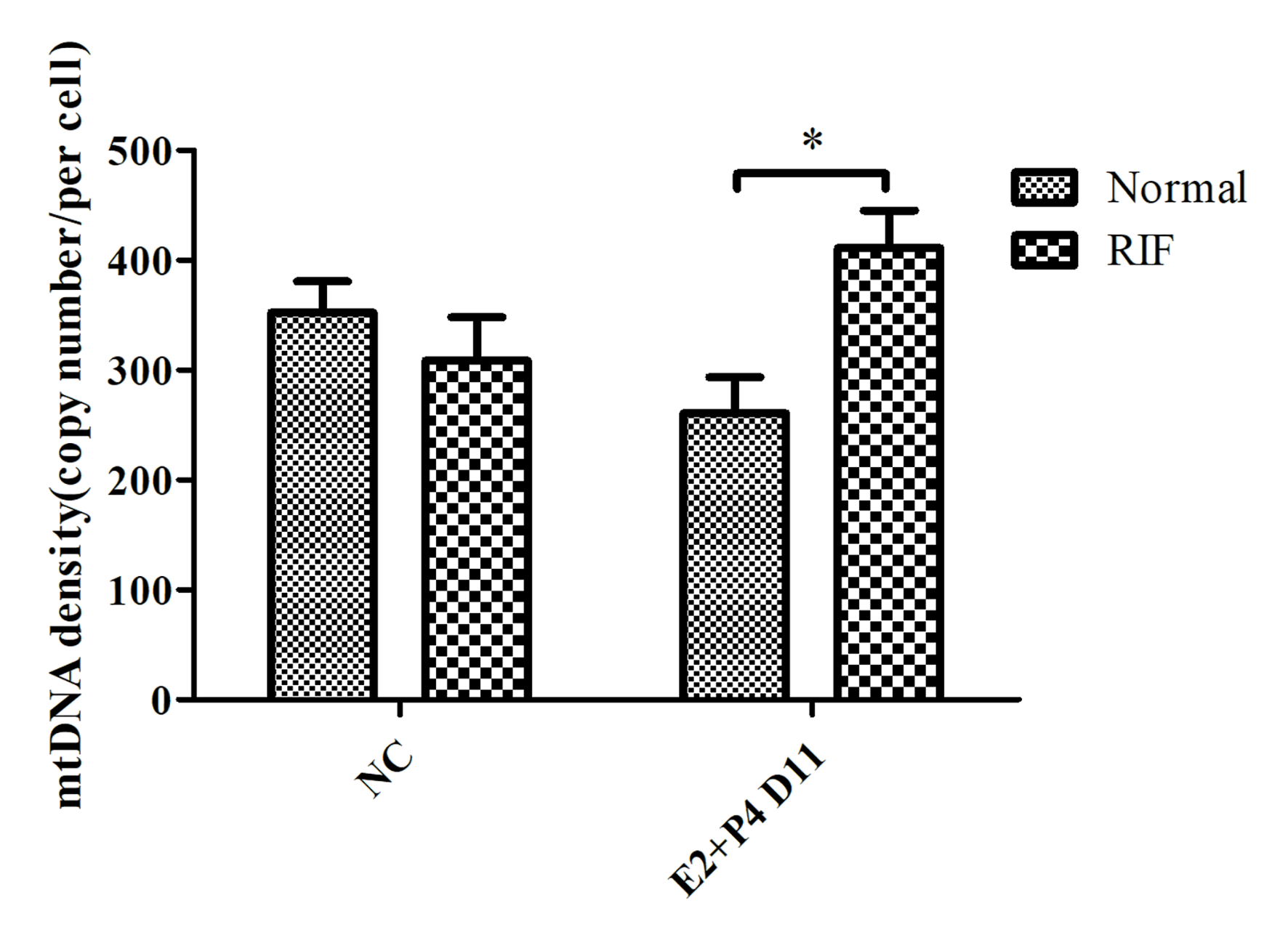

Supplement: Supplementary file 8 — Figure S8. RIF organoid showed an increased number of mitochondrial DNA copy. p < 0.05. [file CPR-58-e13819-s011.tif]

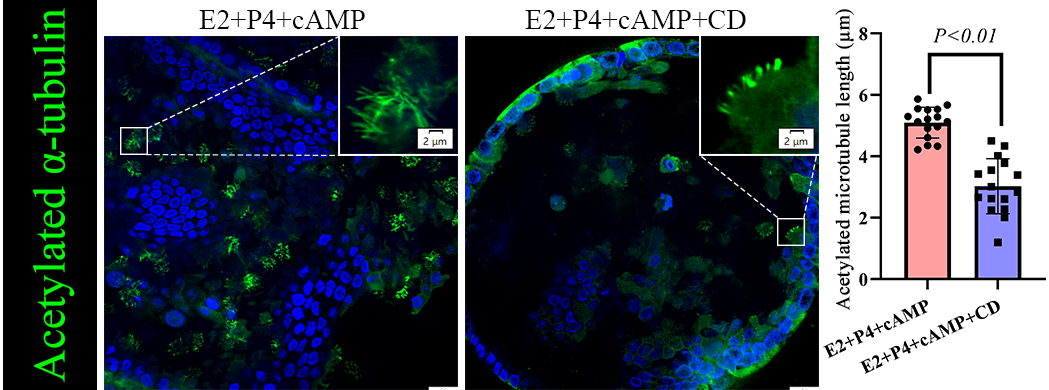

Supplement: Supplementary file 9 — Figure S9. Ciliobrevin D induced ciliary injury. Cilia‐specific antagonist ciliobrevin D treatment induced ciliary acetylated microtubule deficiency so that their length was shorter than normal. Scale bars, 20 μm. p < 0.01. [file CPR-58-e13819-s009.tif]

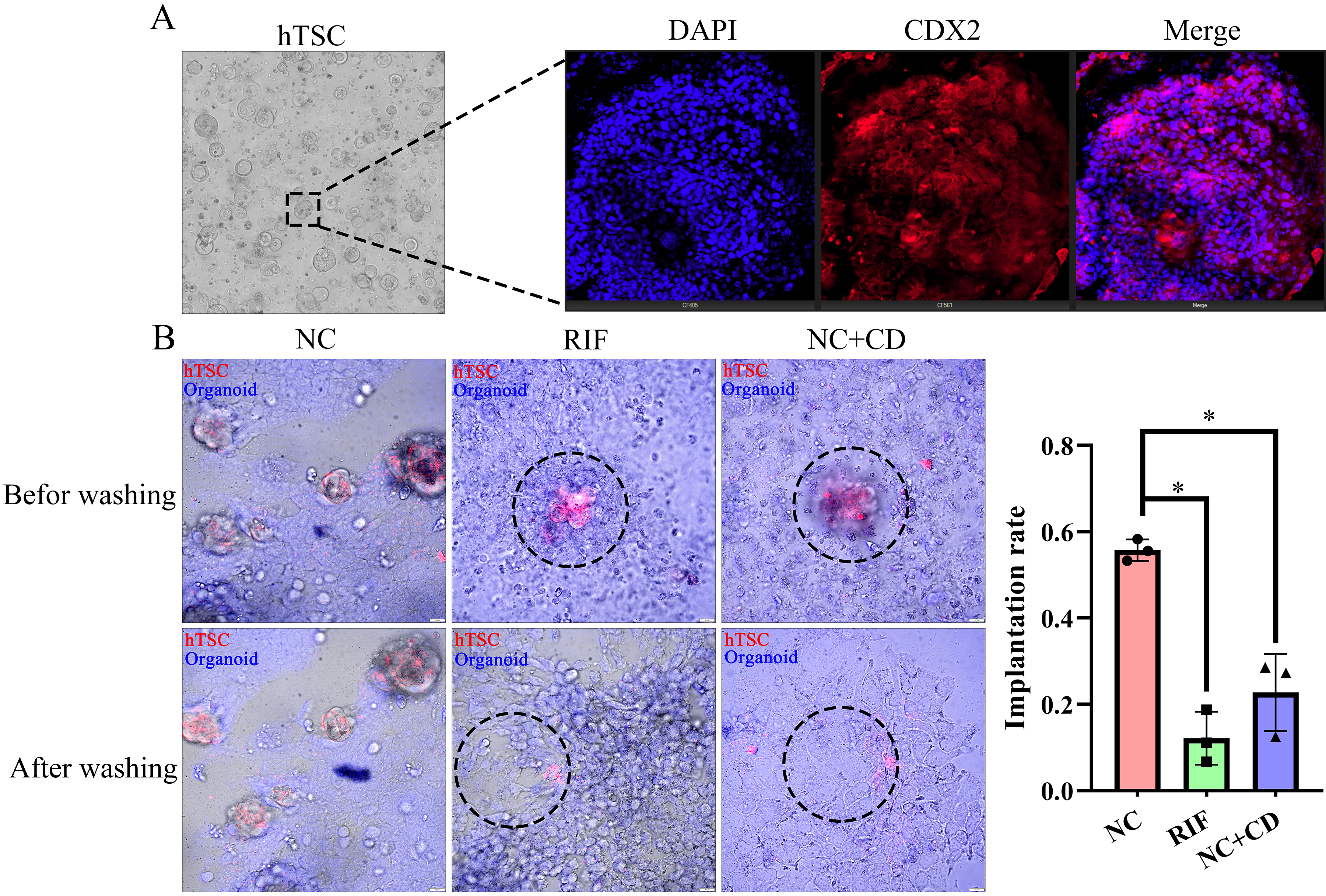

Supplement: Supplementary file 10 — Figure S10. The hTSCs ball cannot adhere to the epithelial cell after anti‐cilia reagent treatment. We set 20 hTSCs balls on the surface of the cell layer, and check it after 24 h. Compared with more than 10 balls firmly adhesion in the normal group, hTSCs balls got loose adhesion in the ciliobrevin D treated and RIF group and easily washed away by pipette. After washing, the rest balls number divide by 20 to get the final adhesion rate. Scale bars, 100 μm. p < 0.05. [file CPR-58-e13819-s004.tif]

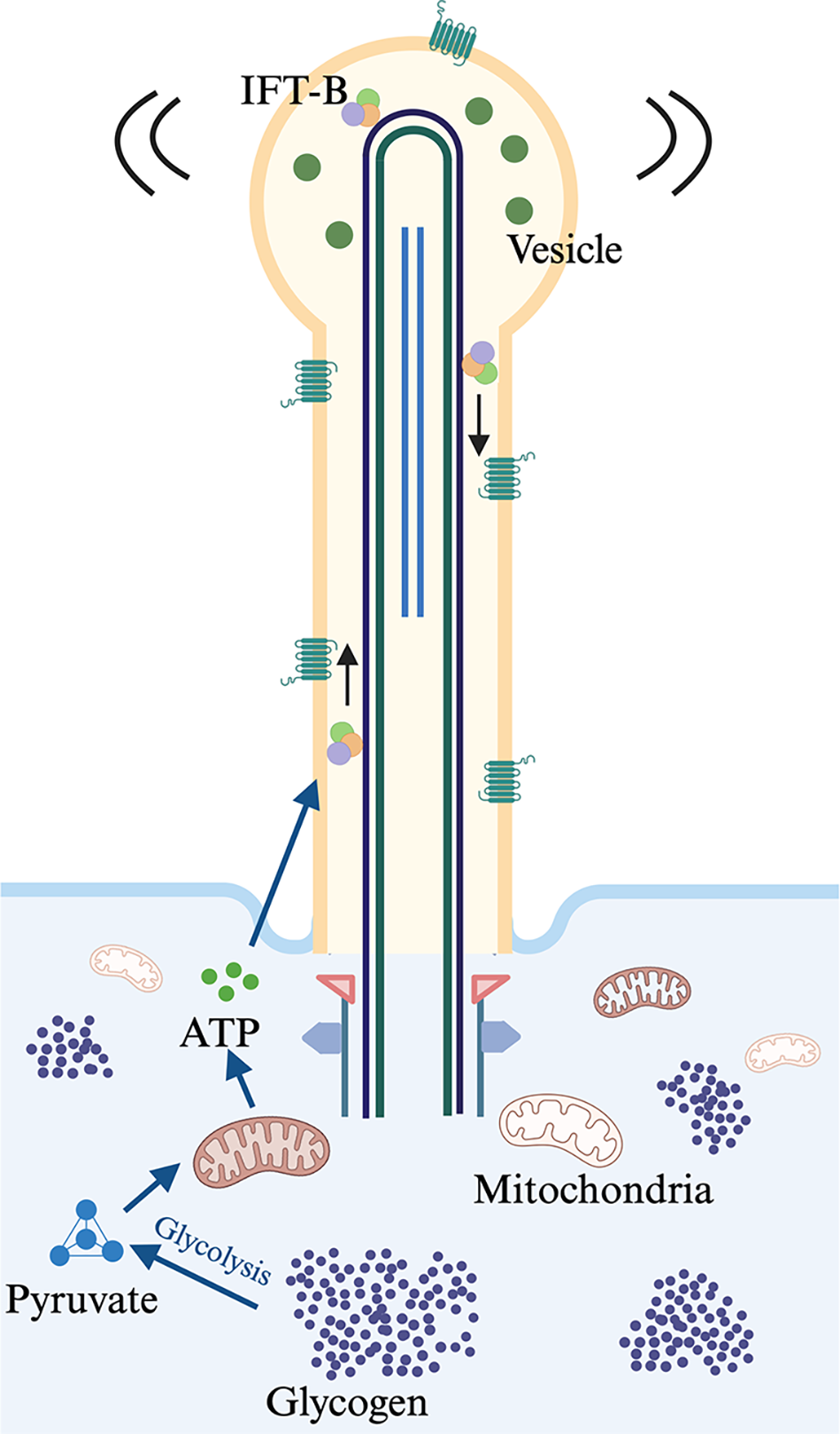

Supplement: Supplementary file 11 — Figure S11. Mitochondria‐accelerated motile cilia intraflagellar transport supplied by glycogenesis and glycolysis energy may be involved in endometrial microenvironment establishment. [file CPR-58-e13819-s002.tif]
