# Supplementary material for: Ciliary IFT‐B Transportation Plays an Important Role in Human Endometrial Receptivity Establishment and is Disrupted in Recurrent Implantation Failure Patients
Source: Cell Prolif. 2025 Feb 6;58(7):e13819. doi: 10.1111/cpr.13819 (PMC12240636; doi:10.1111/cpr.13819)
Supplement: Supplementary file 18 — Data S1. [file CPR-58-e13819-s014.docx]

**Methods Details**

**1 Endometrial ogranoid establishment and confirmation.**

Endometrial tissue biopsies were immediately placed in cooled normal saline supplemented with 1% antibiotic-antimycotic (Gibco, 15240062, USA) or cooled phenol red-free DMEM/F12 (Gibco, 11039021, USA) supplemented with 1% antibiotic-antimycotic and 10% charcoal-stripped FBS (BI, 042011A, Israel) and transferred to the laboratory within 1 h. The biopsies were subsequently rinsed once with a solution containing 0.01% benzalkonium bromide and then washed three times with phenol red-free DMEM/F12 supplemented with 1% antibiotic-antimycotic. The tissues were placed in 100 mm petri dishes with 10 ml of digestion solution (cooled PBS with 0.1% collagenase IV [Gibco, 17104019, USA]) and cut into 1 mm^3^ pieces. This mixture was placed in an incubator at 37 °C and 5% CO_2_ for 40 minutes. After digestion, the same volume of neutralizing medium (phenol red-free DMEM/F12 supplemented with 1% antibiotic-antimycotic and 10% charcoal-stripped FBS) was added to stop digestion, and then we used a 1 ml pipette to aspirate the mixture and separate the epithelium. The mixture was filtered through a 70 μm cell strainer (Falcon, 431751, USA), undigested tissue fragments were blocked at the bottom of the strainer, and stromal cells and epithelial cells were collected in a 50 ml centrifuge tube. Then, the filtered solution was passed through a 40 μm strainer to separate stromal cells and epithelial cells; the epithelial cells passed through the bottom of the membrane. The strainer was inverted, placed on a new centrifuge tube and rinsed with 6 ml of phenol red-free DMEM/F12 supplemented with 1% antibiotic-antimycotic and 10% charcoal-stripped FBS. Then, the solution containing endometrial epithelial cells was collected in a 50 ml centrifuge tube. After centrifugation (1500 r, 10 min), the supernatant was removed, and the sediment was resuspended in 1 ml of advanced DMEM/F12 medium (Gibco, 12634010, USA). After the supernatant was removed, the sediment was resuspended in Matrigel (Corning, 356231, USA) and placed on ice. Thirty-microliter droplets of Matrigel cell suspension were added to a 24-well plate (1 droplet per well) and incubated at 37 °C for 15 min.

Endometrial tissues were derived from three people. Then, epithelial cells were isolated from the endometrium in the secretory phase and cultured in Matrigel after enzymatic digestion. The cells spontaneously formed a sphere with an empty chamber after 4 days (Fig. S1A, B). After 3 passages, the cells were found to be epithelial, as confirmed by CK18 immunostaining, and we found obvious expression of MUC1, LIF, and CDH1, markers of endometrial epithelial cells (Fig. S1C), showing that these organoids contained characteristic endometrial features. As we hypothesized, these organoids were able to regenerate from single cells after digestion and achieve more than 14 passages.

**2 Hormones stimulation of organoid**

As presented before[1, 2], we chose estrogen and progesterone combined stimulation to induce epithelial cells from proliferating phase to secretory phase. Organoids were treated with either 10 nM estradiol (E2, Sigma, E1024) or vehicle as a control (100% ethanol) for 2 d. Following this, organoids were treated with either 10 nM E2 and 10 nM E2, 1 μM P4 (Sigma, PHR1589), and 1 μM cAMP (2′-Odibutyryladenosine 3′, 5′-cyclic monophosphate sodium salt; Sigma, D0627) (E2+P4+cAMP) for a further 4d, 5d, and 6 d. And samples were collected from each time site. And change fresh culture medium every two days.

And hormones preparation list as below sheet.

| **Name** | **Company** | **Cat.** | **Stock concentration** | **Work concentration** |
| --- | --- | --- | --- | --- |
| Estradiol | Sigma | E1024 | 100mM (0.02724g in 1ml 100% ethanol) | 10 nM |
| Progesterone | Sigma | PHR1589 | 100mM (0.0315g in 1ml 100% ethanol) | 1 μM |
| 2′-O’dibutyryladenosine 3′,5′-cyclic monophosphate sodium salt; | Sigma | D0627 | 1mM (0.43008mg in 100% ethanol) | 1 μM |

**3 RNA-Sequencing**

Organoids recovered from Matrigel by cell recovery solution, and stored at -80℃ refrigerator. Total RNA was isolated by Trizol (Ambion company, USA) immediately after sample collected from operating room and cDNA synthesis was performed using Takara Bios RR047A complementary DNA (cDNA) Synthesis kit. Sequencing libraries were generated and sequenced by Novogene (Beijing, China). The primary library was constructed by total RNA (amount≥1μg). mRNA contained polyA tail was enriched by Oligo(dT) magnetic bead and randomly broken up in Fragmentation Buffer. The first string of cDNA was synthesized using M-MuLV reverse transcriptase system, and the second string was synthesized using DNA polymerase I system. The 370~420bp double-stranded cDNA was scanned and purified by AMPure XP beads after PCR amplification, and finally formed library. The library’s primary quantification was done by using Qubit 2.0 Fluorometer. The insert size was detected by Agilent 2100 bioanalyzer (library concentration diluted to 1.5ng/μl). RNA-Sequencing was processed on the Illumina platform after library inspection. The reads of paired ends were compared with reference genome established by HISAT v2.0.5. FeatureCounts(1.5.0-p3) were used to calculate the reads of each gene. The fragments per kilobase per million (FPKM) mapped reads of gene was calculated based on the length and mapped reads. DESeq2 software (1.20.0) was used to detect DEGs between the two groups (two replicates for each group). Negative binomial distribution was used to determine differential gene expression. P-value was obtained by Benjamini and Hochberg method. DEG analysis was calculated by edgeR (3.22.5). Gene expressions with a P value of <0.01 and |log2foldchange|≥2 were considered as differentially expressed genes. DEG analysis was performed by using clusterProfiler (3.4.4) software. A gene ontology (GO) term with a calibrated P-value lower than 0.05 was selected as a different expression enrichment item.. Biological process, cellular component and molecular functions were analyzed by clusterProfiler in KEGG pathway.

**4 Proteomics**

**4.1 Protein collection**

Solution preparation:

Lysate (4% SDS 50mM Hepes PH=8) : Weigh 476.6mg Hepes, add 40mL ultra-pure water, fully dissolve, adjust the pH to about 8.0 with NaOH on the pH meter, then weigh 1.6g SDS and add it to make the SDS fully dissolve. Cocktail protease inhibitors were added at 1:1000 by volume, and phosphorylase inhibitors were added at 1:100 (when the enzyme inhibitors were used, they were added for immediate use), mixed, and stored at room temperature.

a. Organoid collection as described before.

b. The organoids were collected into a 15mL centrifuge tube for 1000g and centrifuged for 5min. Discard the supernatant, add 2mL PBS to it for re-suspension, 1000g, 5min and repeat 3-4 times.

c. Take the centrifuged cells (ice) and add the lysate (same volume to 10^7^cell -- 10cm dish length up to 80% -- 1mg protein -- 500-700μL lysate) to them.

d. Ultrasonic destruction of nucleic acid (ultrasonic 5s, ultrasonic interval 30s, total time 10min, ultrasonic power 20%), the centrifuge tube is inserted on the ice, until the solution is no longer sticky.

f, 14000g, 10 min centrifuge, the supernatant is the protein solution, transfer it to a new centrifuge tube, stored it at -80℃.

g, BCA method was used to determine protein molecular concentration.

**4.2 SDS-PAGE detect protein content**

(1) Solution configuration:

a. 10%APS: Weigh 0.1g APS, add 1mL deionized water, dissolve fully, store at -20℃.

b. SDS-PAGE gel: add propionamide, APS, water, gel buffer, TEMED respectively in accordance with the proportion, after adding TEMED gel immediately solidified. After the separation gel has solidified, pour the concentrated gel.

c. Loading buffer;

d. Coomassie bright blue dye:

e. Decolorizing solution: methanol, glacial acetic acid, water according to the volume ratio of 1:1:8 ratio mixed evenly.

(2) Operation process:

Taking 20ug of protein for electrophoresis. And decolorization, the early 5-10min to change a decolorization solution, change 3-4 times, the late half an hour to replace a decolorization solution, cycle 4-5 times, can be removed within 4h.

**4.3 Reductive alkylation, acetone precipitation**

(1) Solution preparation:

a. 500mM Hepes pH=8: The preparation method is the same as the above.

b. 800mM CAA(light-proof preparation) : Weigh 74.808mg CAA in a light-proof centrifuge tube, add 1 mL 50mM Hepes(PH=8.0), mix well and dissolve fully, and place away from light.

c. ice acetone: measure 10 mL acetone and put it in the refrigerator at -20 ℃.

d. 75% ice acetone: Take 7.5mL acetone and add 2.5mL ultra-pure water to the refrigerator at -20℃.

(2) Operation process:

a. According to the determination of protein concentration, take the appropriate quality of protein (one IBT/TMT reagent to label up to 100μg protein) into 1.5mL centrifuge tube, and use tin foil to avoid light.

b. Reduce alkylation, add appropriate [V(TCEP)=V(protein)/49] volume of TCEP and appropriate [V(CAA)=V(protein)/39] volume of CAA, TCEP acting concentration is 10mM, CAA acting concentration is 20mM, add reagents and mix, at 37℃, 800 rpm, on the mixer. 1h reaction.

c. after the reaction is completed, add 5 times the volume of ice acetone, overnight precipitation protein at -20℃ refrigerator, the following steps can also be carried out for more than 4 hours.

**4.4 Proteolysis**

(1) Solution preparation:

a. 50 mM Hepes: The configuration method is the same as the above.

b. 8M urea (50mM Hepes) : Weigh 480.48mg urea, fill the volume with 50mM Hepes to 1mL, shake well (urea swelling phenomenon, do not directly add 5mL 50mM Hepes).

c. 200mM TEAB: Take 1mL TEAB, add 4mL ultra-pure water, and dilute it to 200mM.

d. 8M urea (200mM TEAB) : weigh 480.48mg urea, fill 200mM TEAB volume to 1mL, shake well (urea swelling phenomenon, do not directly add 5mL 50mM Hepes).

e. 0.1% TFA(pH= about 2) : First, 10% TFA solution (take 900uL ultra-pure water from the fume hood into 1.5mL centrifuge tube, then take about 100uL TFA(strong acid, volatile, can take more) and mix), then take 990uL ultra-pure water, add 10uL 10% TFA, and mix.

f. Trypsin liquid: Put trypsin on ice, quickly weigh 1mg trypsin, add 1mL 0.1%TFA to make 1μg/μL trypsin liquid, 200μL in one portion, can be stored in the refrigerator at -80℃ for a long time.

(2) Operation process:

a. Take acetone overnight precipitated protein, 4 ℃, 14000g, 20 min, centrifuge to remove acetone, use a gun to absorb the acetone, do not pour the acetone directly. Then 500 ul 75% acetone was added to clean the precipitated protein, the acetone was removed by centrifugation at 4℃, 14000 g, 10 min, and the protein sample was put on ice to evaporate the remaining acetone.

b. unlabeled proteolytic hydrolysis:

b1. Dissolve the protein with 8M urea (50mM Hepes), change the protein concentration to 5μg/μL, absorb and mix the dissolved protein. Check whether the protein is completely dissolved by high speed centrifugation (14000g).

b2. dilute the protein solution to prevent high concentration of urea from inhibiting trypsin activity, add 50mM Hepes(pH=8.0) to make the protein solution become 0.5μg/μL(10 times dilution, if the volume is too large can be diluted eight times), and the urea concentration is reduced to less than 2M. High speed centrifugation (14000g) was used to check whether the protein was completely dissolved.

c. labeled proteolytic hydrolysis:

Dissolve 100μg protein with 10μL 8M urea (200mM TEAB), mix well and dissolve fully, check whether the protein is completely dissolved by high-speed centrifugation (14000g), then add 90μL 200mM TEAB(tenfold dilution of urea), and check whether the protein is completely dissolved by high-speed centrifugation (14000g).

d. Add trypsin solution to the dissolved protein solution so that the mass ratio of protein and enzyme is 50:1.

e. Mix the solution with pancreatic enzyme, centrifuge it, and place it in a metal bath at 37℃, 800rpm, and oscillate the enzymolysis overnight for >12h.

f. After enzymatic hydrolysis overnight, heat at 100℃ for 5min to inactivate trypsin.

**4.5 Protein quantitative labeling**

(1) Solution preparation:

a. IBT/TMT dissolution: Take out the IBT reagent in the refrigerator at -80℃, place it at room temperature for 10min, first 1000g, centrifuge for 30s, and then add 52μL anhydrous acetonitrile. Eddy dissolves for 5min.

b. 5% hydroxylamine solution: Take 50uL hydroxylamine solution and add 250uL ultra-pure water.

(2) Operation process:

a. Taking the enzymolized peptide, add IBT labeling reagent according to the mass ratio of 1:5, shake, mix and react at room temperature for 2h.

b. Two hours later, 10μL 5% hydroxylamine was added to each sample for 30min, and the labeling reaction was terminated.

c. Placing the sample in the condensing concentrator and spin dry the liquid.

**4.6 Peptide desalt**

(1) Solution preparation:

a. 100% acetonitrile: Take 10mL acetonitrile.

b. 10%TFA: Take 100uL TFA out of the fume hood, add 900uL ultra-pure water into it, and mix well.

c. 50% acetonitrile 0.1%TFA: Add 20mL acetonitrile, 400μL 10% TFA, 19.6mL ultra-pure water into 40mL centrifuge tube successively, and mix well.

d, 0.1%TFA: Take 400μL 10%TFA solution and add 39.6ml ultra-pure water.

(2) Operation process:

a. Take the dried peptide, add 900uL 0.1%TFA solution to it, dissolve the peptide for 1min by ultrasound, then add 90uL 10% TFA, dissolve the peptide for 1min by ultrasound, and put it on ice for 20 min.

b. The acidified peptide sample was centrifuged at 4℃, 14000g, for 10min, and the supernatant (the bottom is undigested protein) was taken.

c. activate the C18 column (if activated small column, all volumes changed to 200ul). The C18 column was washed once with 1mL pure acetonitrile, then washed twice with 1mL 50% acetonitrile 0.1%TFA, and then washed three times with 1mL 0.1%TFA.

d. After the new collection tube is replaced, the sample is passed through the C18 column, each C18 column can elute the protein sample 1-3mg, and the sample is repeated through the column 3 times

e. Add 1mL 0.1% TFA, eluate the salt in it, and wash it repeatedly three times.

f. replace the collection tube, add 300μL 50% acetonitrile, elute the protein on the column, repeat elute twice, elute 600ul.

g. Mix the eluted peptide and then pack it, condense and dry it on the condenser.

**4.7 Peptide fractionation**

HIGH pH REVERSE PHASE SEPARATION

The peptide mixture was re-dissovled in the buffer A (buffer A: 20 mM ammonium formate in water, pH 10.0, adjusted with ammonium hydroxide), and then fractionated by high pH separation using Ultimate 3000 system (ThermoFisher scientific, MA, USA) connected to a reverse phase column (XBridge C18 column, 4.6 mm x 250 mm, 5 μm, (Waters Corporation, MA, USA). High pH separation was performed using a linear gradient, starting from 5% B to 45% B in 40 min (B: 20mM ammonium formate in 80% ACN, pH 10.0, adjusted with ammonium hydroxide). The column was re-equilibrated at the initial condition for 15 min. The column flow rate was maintained at 1 mL/min and the column temperature was maintained at 30℃. Twelve fractions were collected; each fraction was dried in a vacuum concentrator for the next step.

**4.8 Preparation of peptide sample**

(1) Solution preparation:

a, A: 50% acetonitrile, take 5mL acetonitrile, add 10uL of formic acid, then add 4.99mL of ultra-pure water, mix well.

b, B solution: 0.1% FA, take 9.99mL acetonitrile, add 10uL of formic acid, mix well.

(2) The operation process:

the maximum sample is 20μL, usually 10μL, no more than 1μg

a. Taking out the freeze-dried sample, and redissolve the peptide sample.

b. Adding 50% acetonitrile (double the volume) to the sample and ultrasonic for 1min after vortex.

c. Adding 0.1%FA (9x volume), ultrasonic 1min d after vortex, centrifuge at 6℃, 14000g, 20min.

e. The mass spectrum sample bottle, running.

**4.9 nano-HPLC-MS/MS analysis**

The peptides were re-dissolved in solvent A (A: 0.1% formic acid in water) and analyzed by Orbitrap Fusion coupled to an EASY-nanoLC 1200 system(Thermo Fisher Scientific, MA, USA）. 2μL peptide sample was loaded onto a 25 cm analytical column (75 μm inner diameter, 1.9 μm resin (Dr Maisch)) and separated with 90min-gradient starting at 4% buffer B (80% ACN with 0.1% FA) followed by a stepwise increase to 30% in 80 min, 90% in 2 min and stayed there for 8 min. The column flow rate was maintained at 600 nL/min with the column temperature of 55°C. The electrospray voltage was set to 2 kV.

The mass spectrometer was run under data dependent acquisition (DDA) mode, and automatically switched between MS and MS/MS mode.The survey of full scan MS spectra (m/z 350-1550) was acquired in the Orbitrap with 60,000 resolution. The Normalized automatic gain control (AGC) target of 100% and the maximum injection time of 50ms. Then the precursor ions were selected into collision cell for fragmentation by higher-energy collision dissociation (HCD), the normalized collection energy was 38%. The MS/MS resolution was set at 60,000, the Normalized automatic gain control (AGC) target of 160%, the maximum injection time of 120ms, and dynamic exclusion was 30 seconds.

**4.10 Data Analysis**

The raw Data of DDA were processed and analyzed by SpectroMine3.2 (Biognosys AG, Switzerland) with default settings. The database was uniprot-homo sapiens (version2022, 20610 entries). Trypsin was set as the digestion enzyme. The max missed cleavage was set as 2. Carbamidomethylation on cysteine and TMT 16plex on lysine and the N-term of protein were specified as the fixed modification. Oxidation on methionine was specified as the variable modifications. PSMs were filtered by 1% FDR, peptides were filtered by 1%FDR, proteins were filtered by 1%FDR . Reporter ions were used to calculate the quantification ratio between samples. Normalization was calculated from the total intensity of all labels in all quantifiable peptides. Different expressed proteins were selected if their p value<0.05，FC>1.3,<1/1.3.

**5 Living imaging observation**

Considering these spheres concentrated on the day 11, these organoids treated with combined hormones for 4 days were collected and mixed with fresh Matrigel, and then drop it on the 35mm confocal dish. And then cultured with 500 μl fresh differential culture medium. We use SIR-Tubulin (CY-SC002, Cytoskeleton) to monitor cilia activity, and the Hoechst 33258 (C1011, Beyotime) was used to locate the nucleus, and Mito-Tracker (M7514, Sigma) was used to monitor mitochondria movement. Then, live images were captured by a spinning disk confocal superresolution microscope (Olympus, SpinSR10) after 24 hours of incubation. Setting the program to capture figures every 5s for a total of 10 minutes.

**6** **Ultramicroproteome**

**6.1 Tryptic digestion**

For the digestion process, 30 μL of 0.1 M Ammonium bicarbonate (ABB, GENERAL-REAGENT®, Cat #1066-33-7) and 0.5 μg (1 μL) of trypsin (Hualishi Tech, Cat # HLS TRY001C) was added to each sample, followed by incubation at 37℃ on a shaker set to 220 rpm for 16 hours. To terminate the digestion, 3 μL of 10% Trifluoroacetic acid (TFA, Thermo Fisher Scientific, Cat # 85183) solution was added to each tube, achieving a final TFA concentration of 1%, followed by sample drying and pH verification to ensure a range of 2-3. For mass spectrometry analysis, 10 μL of the peptide solution from each sample was transferred into vials.

**6.2 Proteome sample analysis**

Liquid chromatography-mass spectrometry analysis was conducted using a UHPLC system (Bruker Daltonics, Germany) coupled with a timsTOF Pro mass spectrometer (Bruker Daltonics, Germany) for Data-dependent acquisition (DDA) and label-free quantification. The mobile phase A consisted of 100% water with 0.1% formic acid, and the mobile phase B comprised 100% acetonitrile with 0.1% formic acid. All reagents used were of mass spectrometry grade. During DDA acquisition for all samples, peptides were first loaded onto a pre-column (5 mm × 300 μm i.d.), then introduced into an analytical column (1.9 μm, 120 Å, 150 mm × 75 μm i.d.) at a flow rate of 300 nL/min, analyzed using a 60-minute LC gradient (0-50 min, 5-27% mobile phase B; 50-60 min, 27-40% mobile phase B). The mass spectrometry scanning parameters were as follows: The TIMS accumulation time was set to 2 ms, with the PASEF MS mass scan range spanning 100-1700 m/z and a 1/k0 scan range of 0.6-1.6. The signal detection threshold was set at 5,000. The PASEF CID energy ranged from 20 eV to 59 eV, with 10 PASEF MS/MS scans performed. The charge range was 0-5, and the signal detection threshold for MS/MS was set at 2,500 cts/s.

**7 RT-qPCR and digital PCR**

The experiments were done as previously described[32]. The specific RT-PCR primers showed on Table S1. For relative mRNA expression analysis, we performed real-time RT-qPCR assay using CFX 96TM Real-Time PCR Detection System (Bio-Rad, Hercules, CA, USA). Real-time RT-qPCR assay was performed using the Takara RR820A kit (Takara, Japan) as per the manual instructions and our laboratory practice. For this purpose, we used a 10-μL reaction mixture containing 1 μL cDNA, 5 μL TB Green Premix Ex Taq II (Takara, Kusatsu, Shiga Prefecture, Japan), 0.5 μL each of the forward and reverse primers, and 3 μL ddH2O. Glyceraldehyde 3-phosphate dehydrogenase (Gapdh) was used as an internal reference gene for normalization. The PCR reaction conditions were as follows: (1) an initial denaturation at 95 °C for 3 min; (2) 40 cycles of 95 °C for 10 s; and (3) primer-specific annealing temperature for 30 s (Table S1). A melting curve analysis was performed to validate the reaction specificity. Only one product of desired size was identified, and a single smooth peak was observed for each primer in melt curve analyses. Each sample was analyzed in triplicate. The relative mRNA expression levels were calculated using 2−ΔΔCt (Livak) method.

In order to detect mitochondria DNA copy changes after hormones stimulation in organoid, we used digital PCR to detect mtDNA difference. Organoid DNA isolation was followed instruction of nucleic acid extraction kit (R00301, Biorain, China) and dPCR chip also purchased from Biorain. We used a 20-μl reaction mixture containing: 10μl 2×AplusTM ddPCR Mix, 1μl each of the forward and reverse primers of the reference and target gene, 0.8μl each of the probes of reference and target gene, 3.4μl ddH2O and 1μl DNA. In this experiment, we set RPP30 as reference gene, and ND1 as target gene of mitochondria. Primers’ sequence were listed in Table S2. The dPCR conditions were as follows: (1) RNase dysfunction at 37°C for 10min, (2) an initial denaturation at 95 °C for 3 min, (3) 40 cycles of 95 °C for 15 s, (4) annealing at a primer-specific annealing temperature for 45 s. Each sample was analyzed in triplicate. Positive drops were captured by DropXpert S6, and according to copy number per cell to detect mtDNA changes. PCR primers listed as follow: RPP30-F: 5’-AACTTGTAAGTGGTAGTGCATAGA-3’/ R: 5’-GTAGGAGGACATTTGAGGAGTG-3’, ND1-F: 5’-CTGATCAGGGTGAGCATCAAA-3’/ R: 5’-GAATGATGGCTAGGGTGACTTC-3’.

**8 Western blots**

After degum, organoids were washed by iced PBS, and then lysed by RIPA (Beyotime, P0013C) for 1hr on ice. Following clarification of the lysate by centrifugation at 15,000 rpm for 15 min. A part of the supernatants were subjected to BCA analysis to determine total protein concentration, the rest of samples were mixed with 5×SDS loading buffer diluted to 1× and incubated at 95℃ for 10min. We added 30μg protein every lane. Proteins were separated in 10% PAGE Gel (Epizyme, PG112) in SDS running buffer, then transferred onto PVDF Transfer membrane (Millipore, IPVH85R) in transferring buffer. Membranes were incubated in 5% skim milk at 4℃ overnight. Next, membranes were washed 3 x 5 min in TBST buffer at room temperature, and then incubated with secondary antibody diluted by blocking buffer for 1hr at room temperature, then washed 3 x 5 min in TBST buffer. Membranes were scanned using a Bio-rad ChemiDocTM Imaging System or exposed to X-ray film in a dark room. We used 30μg protein in every lane for every WB test.

**9 ATP content determination**

8.1 Reagent Preparation

a. Make 1.0 mL of 1X Reaction Buffer by adding 50 µL of 20X Reaction Buffer (Component E) to 950 µL of deionized water (dH2O). This volume will be sufficient to make 1 mL of 10 mM D-luciferin stock solution.

b. Make 1 mL of a 10 mM D-luciferin stock solution by adding 1 mL of 1X Reaction Buffer (prepared in step 1.1) to one vial of D-luciferin (Component A, blue cap). Protect from light until use. The D-luciferin stock solution is reasonably stable for several weeks if stored at ≤–20°C, protected from light.

c. Prepare a 100 mM DTT stock solution by adding 1.62 mL of dH2O to the bottle containing 25 mg of DTT (Component C, black cap). Aliquot into ten 160 µL volumes and store frozen at ≤–20°C. Stock solutions of DTT stored properly are stable for six months to one year. Thawed aliquots should be kept on ice or at 4°C until ready for use.

d. Prepare low-concentration ATP standard solutions by diluting the 5 mM ATP solution (Component D, green cap) in dH2O. The concentrations and volumes to make depend upon the sensitivity and design of the luminometer to be used. Typically, ATP concentrations ranging from 1 nM to 1 µM are appropriate. These dilute solutions are stable for several weeks when stored at ≤–20°C.

8.2 Standard Reaction Solution

a. We suggest combining the components of the reaction as follows to make 10 mL of a standard reaction solution. Adjust the volumes according to particular requirements: 8.9 mL dH2O+ 0.5 mL 20X Reaction Buffer (Component E)+ 0.1 mL 0.1 M DTT (from step 1.3)+ 0.5 mL of 10 mM D-luciferin+ 2.5 µL of firefly luciferase 5 mg/mL stock solution

b. Gently invert the tube to mix, do not vortex.

8.3 Standard Curve

a. Place an appropriate volume of the standard reaction solution in the luminometer and measure the background luminescence.

b. Start the reaction by adding the desired amount of dilute ATP standard solution and read the luminescence. The volume of the dilute ATP standard solution that is added to the standard assay solution should be no more than 10% of the total assay volume.

c. Subtract the background luminescence.

d. Generate a standard curve for a series of ATP concentrations.

8.4 Sample Analysis

a. Follow the directions given in Standard Curve, substituting ATP-containing samples for the ATP standard solutions. The total volume of the experimental sample assays should be equal to that of the ATP standard assays.

b. Calculate the amount of ATP in the experimental samples from the standard curve.

**10 PRL Elisa Kit**

**10.1 Solution preparation**

a. Washing solution preparation: dilute with distilled water 1:20 (Example: add 19 ml of distilled water to 1 ml of concentrated washing solution)

b. Preparation of standard: Take 7 1.5ml centrifuge tubes and mark them as 1/2, 1/4, 1/8, 1/16, 1/32, 1/64, blank. Add 300ul of standard/sample diluent to the first to seventh tubes respectively, Add 300ul of standard solution ( standard concentration ) to the first tube, mix it on a vortex mixer, and then aspirate 300ul with a pipette and transfer it to the second tube., Repeat this dilution, aspirate 300ul from the sixth tube and discard it and the seventh tube is a blank control. (The following concentrations are recommended for the standard curve: 1/2, 1/4, 1/8, 1/161/32, 1/64, blank).

c. Preparation of biotinylated antibody working solution: 20 minutes before use, dilute 100x biotinylated antilbody into 1x working solution with biotinylated antibody diluent, prepare according to the required amount, use on the same day, and discard the rest.

d. Preparation of ABC complex working solution: 20 minutes before use, dilute 100x concentrated ABC complex into 1x working solution with ABC complex diluent, use it on the same day and discard the rest.

**10.2 Testing procedures**

1. Wash the microplate twice before use , and use it immediately after drying it on filter paper.

2. Sample addition: Add 100ul of standard or sample to be tested to each well, mix the reaction plate and place it at 37℃ for 90 minutes, then shake off the liquid in the ELISA plate and print it on filter paper without washing the plate.

3. Add 100ul of 1x biotinylated antibody working solution to each well, mix well and incubate at 37℃ for 60 minutes

4. Wash the plate: Wash the microplate thoroughly three times with washing solution and blot dry on filter paper.

5. Add 100ul of 1x ABC complex working solution to each well , mix well and incubate at 37℃ for 30 minutes.

6. Wash the plate: Wash the reaction plate thoroughly 5 times with washing solution and blot dry on filter paper.

7. Add 90ul of substrate working solution to each well, mix well and react in the dark at 37℃ for 3-20 minutes.

8. Add 50ul of stop solution to each well, mix well, and measure the absorbance at 450nm using an ELIASA within 30 minutes

1. Fitzgerald, H.C., P. Dhakal, S.K. Behura, et al., *Self-renewing endometrial epithelial organoids of the human uterus.* Proc Natl Acad Sci U S A, 2019. **116**(46): p. 23132-23142.

2. Turco, M.Y., L. Gardner, J. Hughes, et al., *Long-term, hormone-responsive organoid cultures of human endometrium in a chemically defined medium.* Nat Cell Biol, 2017. **19**(5): p. 568-577.
